# Variation in Clinical Treatment and Outcomes by Race Among US Veterans Hospitalized With COVID-19

**DOI:** 10.1001/jamanetworkopen.2022.38507

**Published:** 2022-10-25

**Authors:** Alexander D. Castro, Florian B. Mayr, Victor B. Talisa, Obaid S. Shaikh, Saad B. Omer, Sachin Yende, Adeel A. Butt

**Affiliations:** 1 University of Pittsburgh School of Medicine, Pittsburgh, Pennsylvania; 2 VA Pittsburgh Healthcare System, Pittsburgh, Pennsylvania; 3 Clinical Research, Investigation, and Systems Modeling of Acute Illness Center, Department of Critical Care Medicine, University of Pittsburgh, Pittsburgh, Pennsylvania; 4 Division of Gastroenterology, Department of Medicine, University of Pittsburgh, Pittsburgh, Pennsylvania; 5 Institute for Global Health, Yale University, New Haven, Connecticut; 6 Department of Medicine, Weill Cornell Medicine, Doha, Qatar; 7 Department of Population Health Sciences, Weill Cornell Medicine, Doha, Qatar

## Abstract

**Importance:**

Patients from racially and ethnically minoritized populations, such as Black and Hispanic patients, may be less likely to receive evidence-based COVID-19 treatments than White patients, contributing to adverse clinical outcomes.

**Objective:**

To determine whether clinical treatments and outcomes among patients hospitalized with COVID-19 were associated with race.

**Design, Setting, and Participants:**

This retrospective cohort study was conducted in 130 Department of Veterans Affairs Medical Centers (VAMCs) between March 1, 2020, and February 28, 2022, with a 60-day follow-up period until May 1, 2022. Participants included veterans hospitalized with COVID-19. Data were analyzed from May 6 to June 2, 2022.

**Exposures:**

Self-reported race.

**Main Outcomes and Measures:**

Clinical care processes (eg, intensive care unit [ICU] admission; organ support measures, including invasive and noninvasive mechanical ventilation; prone position therapy, and COVID-19–specific medical treatments) were quantified. Clinical outcomes of interest included in-hospital mortality, 60-day mortality, and 30-day readmissions. Outcomes were assessed with multivariable random effects logistic regression models to estimate the association of race with outcomes not attributable to known mediators, such as socioeconomic status and age, while adjusting for potential confounding between outcomes and mediators.

**Results:**

A total of 43 222 veterans (12 135 Black veterans [28.1%]; 31 087 White veterans [71.9%]; 40 717 [94.2%] men) with a median (IQR) age of 71 (62-77) years who were hospitalized with SARS-CoV-2 infection were included. Controlling for site of treatment, Black patients were equally likely to be admitted to the ICU (4806 Black patients [39.6%] vs 13 427 White patients [43.2%]; within-center adjusted odds ratio [aOR], 0.95; 95% CI, 0.88-1.02; *P* = .17). Two-thirds of patients treated with supplemental oxygen or noninvasive or invasive mechanical ventilation also received systemic steroids, but Black veterans were less likely to receive steroids (within-center aOR, 0.88; 95% CI, 0.80-0.96; *P* = .004; between-center aOR, 0.67; 95% CI, 0.48-0.96; *P* = .03). Similarly, Black patients were less likely to receive remdesivir (within-center aOR, 0.89; 95% CI, 0.83-0.95; *P* < .001; between-center aOR, 0.68; 95% CI, 0.47-0.99; *P* = .02) or treatment with immunomodulatory drugs (within-center aOR, 0.77; 95% CI, 0.67-0.87; *P* < .001). After adjusting for patient demographic characteristics, chronic health conditions, severity of acute illness, and receipt of COVID-19–specific treatments, there was no association of Black race with hospital mortality (within-center aOR, 0.98; 95% CI, 0.86-1.10; *P* = .71) or 30-day readmission (within-center aOR, 0.95; 95% CI, 0.88-1.04; *P* = .28).

**Conclusions and Relevance:**

These findings suggest that Black veterans hospitalized with COVID-19 were less likely to be treated with evidence-based COVID-19 treatments, including systemic steroids, remdesivir, and immunomodulatory drugs.

## Key Points

**Question:**

Are there differences in treatment and clinical outcomes of patients hospitalized with COVID-19 associated with race?

**Findings:**

In this cohort study that included 43 222 adult veterans hospitalized with COVID-19, Black veterans had lower odds of receiving COVID-19–specific treatments, including steroids, immunomodulators, and antivirals.

**Meaning:**

These findings suggest that variation in treatment contributes to differences in COVID-19 care between Black and White patients.

## Teaser

This cohort study assesses within- and between-hospital variance in COVID-19 treatment and outcomes associated with race among hospitalized US veterans.

## Introduction

Preexisting inequities in employment, income, housing, and health care access and differences in exposure risk and comorbidity burden have placed minoritized racial and ethnic populations, such as Black and Hispanic individuals, at greater risk for contracting COVID-19 and associated morbidity and mortality.^1,2,3,4^ Several studies have shown a disproportionally higher risk for adverse short-term outcomes after SARS-CoV-2 infection among these populations.^2,5^ However, most of these studies were limited to the early pandemic period, lacked granular clinical data, or did not account for the correlation of outcomes and exposures among patients within the same hospitals.^6^ Particularly during the early stage of the pandemic, some hospitals faced unprecedented capacity challenges and disruptions in routine care while adapting to a sudden influx of patients with a novel respiratory illness.^7^ These circumstances adversely affected the quality of care and likely resulted in deviation from standard admission and discharge practices.^8,9,10,11,12^ The emergency use authorization and subsequent approval of vaccines,^13^ evidence-based COVID-19 treatments,^14,15,16^ and refinements in the delivery of supportive interventions (eg, early vs late initiation of mechanical ventilation) may have contributed to improved outcomes over time.^17,18^ Whether these interventions equally benefited minoritized and nonminoritized populations has not been well studied. A 2022 study by Wiltz et al^19^ suggested that minoritized patients are less likely to receive outpatient treatment with monoclonal antibodies and inpatient treatment with remdesivir and dexamethasone. However, Wiltz et al^19^ did not account for differences in patient demographics, underlying health conditions, and clustering at the hospital level.

In this study, we used a national cohort of veterans with granular clinical data to characterize differences in the treatment and outcomes associated with race among patients hospitalized with COVID-19. We categorized the pandemic into distinct periods based on waves of COVID-19 hospitalizations. We used statistical methods to account for differences in demographic characteristics, chronic health conditions, acute illness severity, outpatient treatments, and care location and performed sensitivity analyses using a 1:1 matched cohort. We hypothesized that processes of care and short- and long-term outcomes would differ by racial group and over time during the SARS-CoV-2 pandemic.

## Methods

This cohort study was approved by the VA Pittsburgh Healthcare System institutional review board with a waiver of the informed consent requirement per VA Common Rule, Title 38 CFR Part 16. This study is reported following the Strengthening the Reporting of Observational Studies in Epidemiology (STROBE) reporting guideline.

### Study Setting

We conducted this study within the Veterans Health Administration (VHA) health care system, the largest integrated health services system in the United States. In response to the SARS-CoV-2 pandemic, the Department of Veterans Affairs (VA) created the VA COVID-19 Shared Data Resource, as a regularly updated database with extensive demographic, clinical, pharmacologic, laboratory, vital signs, and clinical outcomes information derived from multiple validated sources.^20,21,22^ The VA COVID-19 Shared Data Resource contains structured and unstructured information on all veterans with a confirmed diagnosis of SARS-CoV-2 infection within the VHA, including laboratory data, pharmacy data, and receipt of vaccines.

### Study Population and Design

We performed a retrospective analysis of veterans hospitalized with SARS-CoV-2 infection confirmed via laboratory polymerase chain reaction (PCR) testing at 130 VA hospitals between March 1, 2020, and February 28, 2022, with follow-up until May 1, 2022. To compare care processes and outcomes over time, we categorized hospitalizations during the pandemic into 5 distinct intervals (period 1: March 1 to May 17, 2020; period 2: May 18 to September 1, 2020; period 3: September 2, 2020, to June 7, 2021; period 4: June 8 to October 30, 2021; period 5: October 31, 2021, to February 28, 2022) based on peaks and troughs of COVID-19–related hospitalizations and in a more granular fashion by month of hospitalization (Figure). The first 2 intervals (periods 1 and 2) represent the early pandemic (before the availability of vaccines and evidence-based treatments). In contrast, the last 3 intervals represent the ongoing pandemic and are characterized by the evolution of evidence-based treatment strategies, including steroids, remdesivir, and immunomodulatory drugs and vaccines. We included veterans hospitalized within 14 days after a positive test result or a positive test result within 48 hours after admission.^23^ By limiting our cohort to veterans with at least 2 primary care visits in the 18 months preceding vaccine roll-out, we attempted to identify veterans who regularly use the VHA for their health care needs. Comorbidities were defined per the VA COVID-19 Shared Data Resource. We used the Charlson Comorbidity Index (CCI) to quantify preexisting comorbidities (range, 0-30; higher values indicate a higher burden of chronic comorbidities).^20,21,22,24,25^ The Area Deprivation Index (ADI) was used as a marker of socioeconomic deprivation (range within the sample, 20-192; higher values indicate a higher degree of neighborhood-level of deprivation).^26^ We categorized self-reported race into Black and White and ethnicity within race into Hispanic and non-Hispanic.

**Figure.**
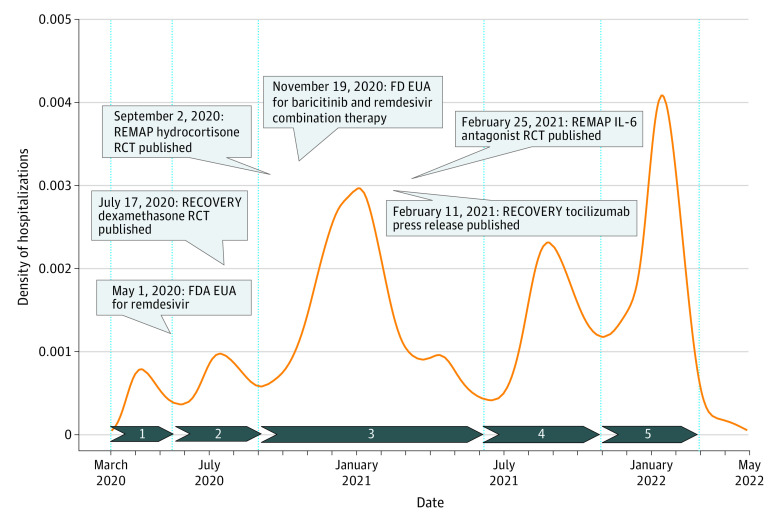
Density Plot of COVID-19–Related Hospitalizations at 130 Department of Veterans Affairs Hospitals Between March 1, 2020, and February 28, 2022 We categorized the pandemic into 5 periods (blue arrows) based on hospitalization peaks and troughs. Period 1 included 1652 patients between March 1 and May 17, 2020; period 2, 3217 patients between May 18 and September 1, 2020; period 3, 17 409 patients between September 2, 2020, and June 7, 2021; period 4, 8690 Patients between June 8 and October 30, 2021; and period 5, 12 254 patients between October 31, 2021, and February 28, 2022. Boxes indicate emerging evidence for COVID-19–specific medical treatments; EUA, emergency use authorization; FDA, US Food and Drug Administration; RCT, randomized clinical trial.

### Outcomes

We quantified clinical care processes, such as intensive care unit (ICU) admission; organ support measures, including invasive and noninvasive mechanical ventilation; and prone position therapy. We compared time from admission to initiation of mechanical ventilation and administration of COVID-19–specific anti-inflammatory treatments between Black and White veterans. We included systemic steroids (dexamethasone and hydrocortisone), antivirals (remdesivir), and immunomodulators (tocilizumab, sarilumab, and baricitinib) in our analyses. Clinical outcomes of interest included in-hospital mortality, 60-day mortality, and 30-day readmissions, including common readmission diagnoses.

### Statistical Analyses

We used descriptive statistics to summarize baseline characteristics and present these as medians with IQRs, means with SDs, and proportions, as appropriate. We compared differences in care processes and outcomes between racial groups and time periods using Wilcoxon rank-sum tests, Kruskal-Wallis 1-way analysis of variance, and χ^2^ tests as indicated. We constructed multivariable generalized linear mixed models to estimate the odds of receiving COVID-19–specific treatments, invasive and noninvasive mechanical ventilation, and experiencing in-hospital death, death within 60 days, and 30-day readmissions. We used a directed acyclic graph (DAG; eFigure 1 in the Supplement) to identify model covariates and potential confounders and included fixed effects for patient demographics (ie, age, sex, ADI, and the proportion of Black patients treated at the hospital), baseline health information (vaccination status, CCI), and information about acute illness severity (time between diagnosis and hospitalization, need for ICU admission, receipt of invasive or noninvasive ventilation, vasopressor support, and kidney replacement therapy). We incorporated random effects for hospital, the proportion of Black patients within hospitals, and the time between diagnosis and hospital admission. As described in prior work, we used the decomposition approach for each model.^6,27^ The within-hospital adjusted odds ratio (aOR) measures the association of the exposure variable race and outcome variable within a given hospital, whereas the between-hospital aOR reflects the association between the proportion of Black patients and the outcome of interest by comparing a hypothetical hospital that exclusively serves Black patients with a hospital that serves no Black patients.^6^

We performed sensitivity analyses and compared the same processes of care and clinical outcomes using a 1:1 matched cohort. Using random coarsened exact matching, we matched White veterans to Black veterans on age (10-year age categories), sex, ADI, CCI category, preexisting chronic kidney and liver disease, ICU admission, mechanical ventilation, oxygen requirement, vaccination status, week of hospitalization, and VHA facility.

We tested the hypotheses that care processes and clinical outcomes varied by race within and between hospitals. We assessed the statistical significance of the differences within and between hospitals from each model by calculating the asymptotic variance of the difference and the corresponding 95% CI. We report within- and between-hospital differences

A 2-sided *P* < .05 was considered statistically significant. We did not adjust for multiple comparisons. We performed all analyses using R statistical software version 4.1.5 (R Project for Statistical Computing). Data were analyzed May 6 to June 2, 2022.

## Results

### Patient Characteristics

We identified 43 222 veterans (12 135 Black veterans [28.1%]; 31 087 White veterans [71.9%]; 40 717 [94.2%] men) with PCR-confirmed SARS-CoV-2 infection hospitalized between March 1, 2020, and February 28, 2022. The median (IQR) age was 71 (62-77) years, and 2955 patients (8.9%) identified Hispanic ethnicity (Table 1).

**Table 1.** Patient Characteristics Stratified by Race and COVID-19 Periods

Characteristic	Patients, No. (%)					
	All periods (N = 43 222)		Periods 1-2 (n = 4869)^a^		Period 3-5 (n = 38 353)^b^	
	Black	White	Black	White	Black	White
No. (%)	12 135 (28.1)	31 087 (71.9)	2192 (45.0)	2677 (55.0)	9943 (25.9)	28 410 (74.1)
Age, median, (IQR), y	67 (59-74)	72 (64-78)	68 (60-74)	72 (64-79)	67 (59-74)	72 (64-78)
Sex						
Men	11 088 (91.4)	29 629 (95.3)	2041 (93.1)	2547 (95.1)	9047 (91.0)	27 082 (95.3)
Women	1047 (8.6)	1458 (4.7)	151 (6.9)	130 (4.9)	896 (9.0)	1328 (4.7)
Ethnicity						
Hispanic	197 (1.6)	2758 (8.9)	52 (2.4)	413 (15.4)	145 (1.5)	2345 (8.3)
Non-Hispanic	11 537 (95.1)	27 201 (87.5)	2097 (95.7)	2206 (82.4)	9440 (94.9)	24 995 (88.0)
Unknown	401 (3.3)	1128 (3.6)	43 (2.0)	58 (2.2)	358 (3.6)	1070 (3.8)
ADI, median,(IQR)	96 (89-104)	93 (85-102)	97 (89-107)	93 (85-101)	96 (89-104)	93 (85-102)
Vaccination status						
None	8833 (72.8)	21 828 (70.2)	2192 (100)	2677 (100)	6642 (66.8)	19 151 (67.4)
Partially	491 (4.0)	1091 (3.5)	NA	NA	491 (4.9)	1091 (3.8)
Fully	2059 (17.0)	6358 (20.5)	NA	NA	2.059 (20.7)	6358 (22.4)
Boosted	752 (6.2)	1810 (5.8)	NA	NA	752 (7.6)	1810 (6.4)
CCI, median (IQR)	3 (1-5)	3 (1-5)	4 (2-6)	3 (2-6)	3 (1-5)	2 (1-5)
Comorbidities						
Coronary artery disease	3140 (25.9)	11 234 (36.1)	529 (24.1)	972 (36.3)	2611 (26.3)	10 262 (35.9)
Malignant neoplasm	2762 (22.8)	6985 (22.5)	482 (22.0)	522 (19.5)	2280 (22.9)	6463 (19.4)
Chronic kidney disease	4030 (33.2)	8134 (26.2)	694 (31.7)	704 (26.3)	3336 (33.6)	7430 (25.0)
Chronic liver disease	1169 (9.6)	3322 (10.7)	192 (8.8)	256 (9.6)	977 (9.8)	3066 (10.8)
Chronic obstructive pulmonary disease	2781 (22.9)	9415 (30.3)	514 (23.4)	767 (28.7)	2267 (22.8)	8648 (28.3)
Diabetes	6303 (51.9)	14 889 (47.9)	1180 (53.8)	1331 (49.7)	5123 (51.5)	13 558 (49.2)
Hypertension	10 046 (82.8)	24 478 (78.7)	1802 (82.2)	2078 (77.6)	8244 (82.9)	22 400 (77.3)
ICU admission	4806 (39.6)	13 427 (43.2)	966 (44.1)	1279 (47.8)	3840 (38.6)	12 148 (42.8)
ICU LOS, median (IQR), d	5 (2-10)	5 (2-10)	6 (2-12)	6 (3-13)	5 (2-9)	5 (2-10)
Hospital LOS, median (IQR), d	5 (2-10)	5 (2-10)	6 (3-12)	6 (3-13)	5 (2-10)	5 (2-10)

Abbreviations: ADI, Area Deprivation Index; CCI, Charlson Comorbidity Index; ICU, intensive care unit; LOS, length of stay; NA, not applicable.

^a^ Defined as March 1, 2020, to September 1, 2020.

^b^ Defined as September 2, 2020, to February 28, 2022.

During the first 2 periods between March 1 and September 1, 2020, 4869 patients (11.3% of the total sample) were hospitalized. Most patients (38 353 patients [88.7%]) were hospitalized during the latter 3 periods, which included the Delta- and Omicron-predominant periods (periods 4 and 5, respectively). Most patients hospitalized during period 1 were Black; however, the proportion of White patients increased over time (Table 1; eTable 1 in the Supplement). The median (IQR) age was lower in Black patients (67 [59-74] years) compared with White patients (72 [64-78] years; *P* < .001) and varied by pandemic period, with the youngest patients hospitalized during periods 2 and 4 (Table 1; eTable 1 in the Supplement). Black patients had higher ADI (median [IQR], 96 [89-104]) compared with White patients (median [IQR], 93 [85-102]; *P* < .001). Black and White patients had a similar burden of preexisting health conditions (median [IQR] CCI for both, 3 [1-5]), and the CCI decreased throughout the pandemic (median [IQR]: period 1, 4 [2-6]; period 5: 2 [1-4]; *P* < .001). Individual comorbidities varied by racial category. For example, coronary artery and chronic obstructive pulmonary disease were more prevalent in White veterans, whereas diabetes, hypertension, and chronic kidney disease were more prevalent among Black patients (Table 1). Most patients in our cohort were not vaccinated (30 661 patients [70.9%]), with a higher proportion among Black patients (8833 patients [72.8%]) than White patients (21 828 patients [70.2%]) and Hispanic patients (2136 patients [72.3%]) than non-Hispanic patients (27 491 patients [71.0%]). As expected, the proportion of patients who were not vaccinated decreased over time after vaccines became available (Table 1; eTable 1 in the Supplement).

### ICU Admission and Respiratory Organ Support

Black patients were as likely to be admitted to the ICU as White patients (4806 Black patients [39.6%] vs 13 427 White patients [43.2%]; within-center adjusted aOR, 0.95; 95% CI, 0.88-1.02; *P* = .17) (Table 2 and Table 3; eTables 6-15 in the Supplement). However, invasive mechanical ventilation was more likely in hospitals that served a higher proportion of Black veterans (between-center aOR, 1.64, 95% CI, 1.02-2.62; *P* = .04). Conversely, Black veterans were less likely to receive noninvasive ventilation (within-center aOR, 0.82; 95% CI, 0.76-0.89; *P* < .001), and it was less likely in hospitals serving a higher proportion of Black veterans (between-center aOR, 0.72; 95% CI, 0.54-0.98; *P* = .03). The duration of mechanical ventilation, time from admission to mechanical ventilation, and use of prone positioning were similar between racial groups, but proning was much less likely among hospitals serving more Black veterans (between-hospital aOR, 0.25; 95% CI, 0.12-0.51; *P* < .001). The proportion of patients treated with invasive mechanical ventilation was highest during the first period (322 patients [20.1%]) and lowest during the last period (677 patients [5.5%]; *P* < .001) (eTable 2 in the Supplement).

**Table 2.** COVID-19–Specific Treatments and Organ Support Stratified by Race and Time Periods Among Patients Requiring Any Supplemental Oxygen

Characteristic	Patients requiring supplemental oxygen, No. (%)						
	All Periods		Periods 1-2^a^		Period 3-5^b^	
	Black (n = 8113)	White (n = 22 891)	Black (n = 1628)	White (n = 2005)	Black (n = 6485)	White (n = 20 886)
Oxygen support						
Low-flow oxygen	7719 (63.6)	21 986 (70.7)	1558 (71.1)	1932 (72.2)	6161 (62.0)	20 054 (70.6)
High-flow oxygen	2593 (21.4)	8181 (26.3)	530 (24.2)	725 (27.1)	2063 (20.8)	7456 (26.2)
MV						
Noninvasive	2329 (19.2)	7550 (24.3)	409 (18.7)	604 (22.6)	1920 (19.3)	6946 (24.4)
Invasive	1057 (22.0)	2597 (19.3)	325 (33.6)	351 (27.4)	732 (19.1)	2246 (18.5)
Total time, median (IQR), d	4 (1-10)	4 (0-11)	6 (2-11)	6 (2-12)	3 (0-10)	4 (0-11)
Time to invasive MV, median (IQR), d	4 (1-10)	4 (1-9)	3 (0-6)	3 (1-8)	5 (1-12)	4 (1-10)
Monoclonal antibodies prior to admission	273 (2.2)	922 (3.0)	NA^c^	NA^c^	273 (2.7)	922 (3.2)
Antivirals prior to admission	17 (0.1)	47 (0.2)	NA^c^	NA^c^	17 (0.2)	47 (0.2)
Received remdesivir						
Any	3767 (46.4)	13 096 (57.2)	228(14.0)	459 (22.9)	3539(54.6)	12 637(60.5)
Time to first dose, median (IQR), d	2 (1-2)	1 (1-2)	2 (1-3)	2 (1-3)	2 (1-2)	1 (1-2)
Received systemic steroids						
Any	4900 (60.4)	16 122 (70.4)	562 (34.5)	899 (44.8)	4338(66.9)	15 223 (72.9)
Time to first dose, median (IQR), d	2 (1-2)	2 (1-2)	2 (1-3)	2 (1-2)	2 (1-2)	2 (1-2)
Received immunomodulators						
Any	562 (6.9)	2343 (10.2)	102 (6.3)	125 (6.2)	460 (7.1)	2218 (10.6)
Time to first dose, median (IQR), d	3 (2-5)	3 (2-4)	5 (3-7)	6 (4-9)	3 (2-4)	2 (2-4)
Prone positioning	425 (40.2)	1298 (50.0)	136 (41.8)	173 (49.3)	316 (43.2)	1125 (50.1)
Extracorporeal membrane oxygenation	NA^c^	11 (0.1)	NA^c^	NA^c^	NA^c^	10 (0.08)
Vasopressors	1262 (26.3)	3152 (23.5)	357 (37.0)	413 (32.3)	905 (23.6)	2739 (22.5)
Kidney replacement therapy	1046 (21.8)	1076 (8.0)	224 (23.2)	128 (10.0)	822 (21.4)	948 (7.8)

Abbreviations: MV, mechanical ventilation; NA, not applicable.

^a^ Defined as March 1, 2020, to September 1, 2020.

^b^ Defined as September 2, 2020, to February 28, 2022.

^c^ Numbers omitted because they were so small as to be irrelevant.

**Table 3.** Odds of Black Veterans Receiving COVID-19–Specific Medical Treatments, Organ Support Measures, and Outcomes Compared With White Patients Stratified by Pandemic Periods

Intervention	Periods 1-2 (n = 4869)^a^				Periods 3-5 (n = 38 353)^b^				All periods (N = 43 222)			
	aOR (95% CI)^c^	*P* value	aOR (95% CI)^d^	*P* value	aOR (95% CI)^c^	*P* value	aOR (95% CI)^d^	*P* value	aOR (95% CI)^c^	*P* value	aOR (95% CI)^d^	*P* value
Medication receipt												
Steroids	1.12 (0.91-1.38)	.29	0.50 (0.23-1.07)	.07	0.85 (0.78-0.94)	.001	0.70 (0.50-0.99)	.04	0.88 (0.80-0.96)	.004	0.67 (0.48-0.96)	.03
Remdesivir	0.80 (0.61-1.06)	.12	0.48 (0.13-1.77)	.27	0.90 (0.84-0.96)	.003	0.70 (0.48-1.02)	.06	0.89 (0.83-0.95)	<.001	0.68 (0.47-0.99)	.02
Immunomodulators	0.84 (0.60-1.20)	.34	0.77 (0.21-2.88)	.70	0.75 (0.66-0.86)	<.001	0.98 (0.61-1.59)	.94	0.77 (0.67-0.87)	<.001	0.97 (0.62-1.53)	.87
Organ support measures												
ICU admission	0.89 (0.74-1.07)	.22	0.51 (0.22-1.21)	.13	0.96 (0.89-1.04)	.33	0.90 (0.51-1.60)	.73	0.95 (0.88-1.02)	.17	0.84 (0.48-1.49)	.55
Invasive mechanical ventilation	1.08 (0.80-1.46)	.60	1.96 (0.84-4.57)	.12	1.10 (0.96-1.26)	.19	1.54 (0.94-2.52)	.08	1.09 (0.97-1.24)	.16	1.64 (1.02-2.62)	.04
Noninvasive mechanical ventilation	0.80 (0.66-0.97)	.02	0.67 (0.36-1.25)	.21	0.82 (0.76-0.89)	<.001	0.72 (0.53-0.97)	.03	0.82 (0.76-0.89)	<.001	0.72 (0.54-0.98)	.03
Proning	0.84 (0.56-1.27)	.41	0.46 (0.12-1.76)	.26	1.07 (0.85-1.35)	.58	0.19 (0.09-0.42)	<.001	1.00 (0.83-1.22)	.98	0.25 (0.12-0.51)	<.001
Outcomes^c^												
Hospital mortality	0.65 (0.49-0.86)	.002	1.14 (0.51-2.59)	.75	1.08 (0.95-1.22)	.24	0.76 (0.50-1.19)	.25	0.98 (0.86-1.10)	.71	0.85 (0.57-1.26)	.42
60-d mortality	0.58 (0.46-0.74)	<.001	1.79 (0.89-3.58)	.10	0.92 (0.84-1.00)	.06	0.75 (0.54-1.04)	.08	0.85 (0.78-0.94)	<.001	0.86 (0.63-1.16)	.32
30-d readmission	0.80 (0.63-1.00)	.05	1.57 (0.82-3.01)	.17	0.98 (0.90-1.06)	.60	1.10 (0.84-1.46)	.48	0.95 (0.88-1.04)	.28	1.14 (0.87-1.50)	.33

Abbreviations: aOR, adjusted odds ratio; ICU, intensive care unit.

^a^ Defined as March 1, 2020, to September 1, 2020.

^b^ Defined as September 2, 2020, to February 28, 2022.

^c^ Within-center aOR. Random effects models were adjusted for age, sex, Charlson Comorbidity Index, Area Deprivation Index, vaccination status, intensive care unit admission, and need for organ support.

^d^ Between-center aOR. Random effects models were adjusted for age, sex, Charlson Comorbidity Index, Area Deprivation Index, vaccination status, ICU admission, need for organ support, and clustering within hospitals. Outcome models were additionally adjusted for COVID-19-specific treatments. Detailed model specifications are provided in eTables 6-15 in the Supplement.

### Anti-inflammatory, Antiviral, and Immunomodulatory Treatments

Approximately two-thirds of patients (21 022 patients [67.8%]) treated with supplemental oxygen or noninvasive or invasive mechanical ventilation also received systemic steroids. The proportion of patients treated with steroids among those treated with supplemental oxygen increased over time (periods 1-2: 1461 patients [40.2%]; periods 3-5: 19 561 patients [71.5%]; *P* < .001) (Table 2; eTable 2 in the Supplement). A more granular illustration of the increase in the proportion of patients treated with steroids, remdesivir, and immunomodulatory drugs over time is illustrated in eFigure 2 in the Supplement. Across all periods, Black patients were less likely to receive steroids (within-center aOR, 0.89; 95% CI, 0.83-0.96; *P* = .004), as were patients admitted to hospitals treating higher proportions of Black patients (between-center aOR, 0.67; 95% CI, 0.48-0.96; *P* = .03) (Table 3). Similarly, across all periods, Black patients were less likely to receive remdesivir treatment (within-center aOR, 0.89; 95% CI, 0.83-0.95; *P* < .001), as were patients admitted to hospitals treating higher proportions of Black patients (between-center aOR, 0.68; 95% CI, 0.47-0.99; *P* = .02) (Table 3). Treatment with immunomodulatory drugs was also less likely for Black patients (within-center aOR, 0.77; 95% CI, 0.67-0.87; *P* < .001).

To understand whether hospital characteristics differed between better and poorer performing hospitals, we plotted the distribution of the within-center odds ratios for steroid treatment by race (eFigure 3 in Supplement) and identified hospitals that were within the bottom and top quintiles. Hospitals in the bottom 20% for within-hospital OR by race were generally smaller and had a higher number of central-line–associated bloodstream infections (CLABSIs) and catheter-related urinary tract infections (CAUTIs). The number of lower complexity and rural hospitals was higher in the 20th percentile group vs 80th percentile group (eTable 5 in the Supplement).

### Clinical Outcomes

#### Mortality

Overall all-cause in-hospital mortality was 4400 of 43 222 patients (10.3%) and declined significantly over time (Table 4; eTable 3 in the Supplement). During the first 2 periods, 772 of 4869 patients (15.9%) died in the hospital, and this rate decreased to 3668 of 38 353 patients (9.6%) during the latter 3 periods (*P* < .001). After adjusting for patient demographic characteristics, chronic health conditions, severity of acute illness, and receipt of COVID-19–specific treatments, there were no differences in hospital mortality between Black and White veterans (aOR, 0.98, CI, 0.86-1.10, *P* = .71), whereas 60-day mortality was lower for Black veterans (aOR, 0.85, CI, 0.78-0.94) (Table 3).

**Table 4.** Clinical Outcomes Stratified by Race and COVID-19 Periods

Outcome	Patients, No. (%)					
	All periods (n = 43 222)		Periods 1-2 (n = 4869)^a^		Period 3-5 (n = 38 353)^b^	
	Black (n = 12 135)	White (n = 31 087)	Black (n = 2192)	White (n = 2677)	Black (n = 9943)	White (n = 28 410)
Hospital mortality	1129 (9.3)	3311 (10.7)	325 (14.8)	447 (16.7)	804 (8.1)	2864 (10.1)
60-d mortality	1818 (15.0)	5695 (18.3)	465 (21.2)	720 (26.9)	1353 (13.6)	4975 (17.5)
30-d readmission						
Any	1534 (13.9)	4063 (14.6)	252 (13.0)	347 (15.6)	1309 (14.3)	3760 (14.7)
Time to readmission, median (IQR), d	9 (4-17)	9 (4-17)	8 (3-16)	9 (4-17)	9 (5-18)	9 (4,-17)

^a^ Defined as March 1, 2020, to September 1, 2020.

^b^ Defined as September 2, 2020, to February 28, 2022.

#### Readmissions

Of 38 782 patients discharged alive, 5597 (14.4%) were readmitted within 30 days. The hospital readmission rate did not differ substantially between early and later pandemic periods (eTable 3 in the Supplement). The median (IQR) time to readmission for Black and White veterans was 9 (4-17) days. The most common readmission diagnoses were COVID-19, sepsis, respiratory failure, diabetes, and atrial fibrillation (eFigure 4 in the Supplement).

### Sensitivity Analyses

Our 1:1 matched validation cohort included 4062 Black and White veterans (eTable 4 in the Supplement). A balance plot for covariates before and after matching is presented in eFigure 5 in the Supplement. Similar to our primary analyses, Black veterans were less likely to receive systemic steroids (1288 patients [63.4%] vs 1351 patients [66.5%]; *P* = .04) or antiviral treatment with remdesivir (1002 patients [49.3%] vs 1107 patients [54.5%]; *P* < .001), and the proportion of patients treated increased over time. For example, the 181 patients (38.7%) needed supplemental oxygen and were treated with steroids during periods 1 and 2, and nearly twice as many patients needed supplemental oxygen and received steroids during periods 3 to 5 (2458 patients [68.4%]). The proportions of patients treated by race and time period are summarized in eTable 4 in the Supplement. In contrast, we did not observe any significant difference in treatment with immunomodulators. Hospital and 60-day mortality were similar in both groups. However, 60-day mortality was lower in Black patients than White during periods 1 and 2 (60 patients [25.5%] vs 80 patients [34.2%]; *P* = .04). The proportion of hospital survivors readmitted by day 30 did not differ by racial group in the matched cohort.

## Discussion

The findings of this cohort study suggest that minoritized populations are less likely to receive COVID-19–specific treatments, including systemic steroids, remdesivir, and immunomodulators. COVID-19 has disproportionately affected minoritized populations, who have experienced higher rates of SARS-CoV-2 infection and COVID-19–related mortality.^1,4,28^ In addition to differences in health care access and exposure risk, differences in quality of COVID-19–specific treatments may contribute to adverse outcomes among minoritized patients.^19^ While the benefit of steroids in COVID-19 has been clearly defined, the evidence for remdesivir and immunomodulatory drugs is less equivocal due to conflicting study results, depending on study populations, severity, and outcome measures.^29,30,31,32^ This persistent equipoise, among many other factors, including structural racism, may contribute to variation in care within and between hospitals.

We have previously shown that Black patients received lower-quality care for community-acquired pneumonia than White patients.^33^ These differences were attributable to differences in case mix and variation in the quality of care between hospitals, and Black patients were more likely to receive care at hospitals that provided lower quality of care in general, regardless of race.

The findings of this study suggests that differences in quality of COVID-19 care are associated with variation in treatment differences within and between hospitals. This apparent discrepancy may be explained by differences in study settings and underlying disease processes. This study was conducted during a pandemic, and we used electronic medical record data to study quality differences in evolving treatments for a relatively homogenous disease caused by a single viral respiratory pathogen. These circumstances may have magnified within- and between-hospital differences in COVID-19 care. In addition, recent reports examining the accuracy of pulse oximetry measurements are concerning for racial bias, suggesting that occult hypoxemia not detected by pulse oximetry occurs far more often in Black patients than White patients.^34,35^ This may result in delayed or missed opportunities to treat otherwise eligible patients with COVID-19,^36^ contributing to within- and between-hospital differences.

Our results confirm recent data from 41 US health care systems participating in the National Patient-Centered Clinical Research Network (PCORNet), which found lower monoclonal antibody treatment use among Asian, Black, and Hispanic patients and patients identifying as another race (eg, American Indian or Alaska Native, Native Hawaiian or other Pacific Islander, or multiple races) SARS-CoV-2 infection compared with non-Hispanic and White patients.^19^ The same PCORNet study reported that Hispanic patients hospitalized with COVID-19 received dexamethasone 6% less often than non-Hispanic inpatients, and Black inpatients received remdesivir 9% more often than White inpatients.^19^ The study did not adjust analyses for differences in baseline patient characteristics (eg, comorbidities) and did not account for clustering within hospitals. Black patients in our cohort were less likely to receive remdesivir than White patients. Our results contradict the assumptions that differences in inpatient treatment by race and ethnicity may be due to differences in clinical indications for medication use based on age and prevalence of specific comorbidities, such as chronic kidney or liver disease, or reflect varying prescribing practices, protocols, and drug access by institutions that serve populations of differential racial and ethnic distributions.^19^ First, the VA issued a COVID-19 response plan,^37^ including specific treatment guidelines and distribution plans, applicable across the entire VA health care system. Second, our models accounted for clustering at the hospital level and preexisting chronic health conditions, including chronic kidney and liver disease. We derived within- and between-center aORs to measure the association between race and outcome variables to account for differences in the proportion of Black patients treated. Third, we performed sensitivity analyses that confirmed our results in a 1:1 matched cohort of Black and White patients matched on demographic characteristics, chronic health conditions, month of hospital admission, vaccination status, ADI, geography, and the severity of COVID-19.

Within-hospital racial differences in the quality of hospital care have been described using various general and surgical patient safety indicators that measure adverse event rates of hospital-acquired illnesses or injuries in a 2021 report by Gangopadhyaya.^38^ Using data from 27 US states, Gangopadhyaya^38^ reported significantly worse patient care for Black vs White patients treated in the same hospitals based on 6 of 11 safety indicators. Despite adjusting for patient characteristics, including insurance coverage type, these differences persisted and were not explained by hospitals that treated the largest proportion of minoritized patients.^38^ These findings are consistent with our results and suggest that differences in the quality of care are due to both within and between hospital differences. We compared hospital characteristics of hospitals that were in the bottom 20% vs the top 20% of the within-center aOR for steroid treatment among Black vs White patients. Hospitals in the bottom 20% were distinctly different from top-performing hospitals regarding size, complexity level, and their association with other health care quality metrics. These quality metrics were also used in a recent study that demonstrated a poorer quality of care for hospitals with higher monthly COVID-19 discharges and hospital size,^12^ suggesting that these routinely collected metrics may serve as a potential surrogate for other care processes in these hospitals.

We did not observe consistent differences in clinical outcomes between Black and White patients. Multiple factors influence clinical outcomes in addition to medical treatments, several of which were unavailable to us (eg, differences in patient treatment preferences or treatment availability, discharge location, code status), all of which could have resulted in residual confounding. In addition, heterogeneity of treatment effect of COVID-19–specific treatments may contribute to the lack of a robust association with survival outcomes. Identifying distinct patient populations who benefit from specific treatments may decrease the variation in care within and between hospitals.

Our study has several strengths, including data from a validated and well-maintained source and a large sample size of patients from 130 hospitals across the US, all within a single integrated health care system.^20^ We adjusted for potential confounding between outcomes and the plausible mediators of the effect of race on outcomes, accounted for clustering within hospitals, and performed additional sensitivity analyses using a 1:1 matched cohort.

### Limitations

Our study has several limitations. First, we performed retrospective analyses utilizing electronic medical record data subject to residual confounding. Second, we did not adjust for hospital bed capacity and subsequent associations with clinical care, patient preferences (eg, refusal of specific treatments), treatment availability in the setting of demand-supply mismatch, and clinical outcomes. Additionally, our cohort consists of a predominantly older, male veteran population in the US, and our results may have limited generalizability to women, younger populations, and nations with different socioeconomic makeup than the US

## Conclusions

In our cohort study, Black race was associated with lower odds of receipt of evidence-based COVID-19 treatments, including systemic steroids, remdesivir, and immunomodulatory drugs. Differences in care were partially explained by within- and between-hospital differences and underscore the need for a comprehension approach to minimize racial variation in COVID-19 care.

## Notes

Supplement. **eTable 1.** Patient Characteristics Stratified by Race and COVID-19 Period **eTable 2.** Treatments and Organ Support Stratified by Race and COVID-19 Period **eTable 3.** Clinical Outcomes Stratified by Race and COVID-19 Period **eTable 4.** COVID-19 Treatments, Organ Support Measures, and Outcomes in a 1:1 Matched Cohort Stratified by Time Periods **eTable 5.** Comparison of Health Care–Associated Infection Rates, Number of Beds, Location, and Complexity Level of Hospitals in the 20th vs 80th Percentile for Within-Center Race Odd Ratios for Steroid Treatment **eTable 6.** Model Specifications and Coefficients for Steroid Treatment **eTable 7.** Model Specifications and Coefficients for Remdesivir Treatment **eTable 8.** Model Specifications and Coefficients for Immunomodulation Treatment **eTable 9.** Model Specifications and Coefficients for Intensive Care Unit Admission **eTable 10.** Model Specifications and Coefficients for Mechanical Ventilation **eTable 11.** Model Specifications and Coefficients or Noninvasive Ventilation **eTable 12.** Model Specifications and Coefficients for Prone Positioning **eTable 13.** Model Specifications and Coefficients for Hospital Death **eTable 14.** Model Specifications and Coefficients for 60-day Death **eTable 15.** Model Specifications and Coefficients for Hospital Readmissions **eFigure 1.** Directed Acyclic Graph (DAG) Illustrating Potential Mediators of the Associations of Race With Treatment Outcomes and Potential Confounders of the Mediators **eFigure 2.** Unadjusted Treatment Outcomes by Race and Month **eFigure 3.** Distribution of Within-Center Adjusted Odds Ratios for Steroid Treatment **eFigure 4.** Most Common Readmission Diagnoses Stratified by Race and Time Period **eFigure 5.** Balance Plot for Covariates Before and After Matching
