# Supplementary material for: Variation in Clinical Treatment and Outcomes by Race Among US Veterans Hospitalized With COVID-19
Source: JAMA Netw Open. 2022 Oct 25;5(10):e2238507. doi: 10.1001/jamanetworkopen.2022.38507 (PMC9597393; doi:10.1001/jamanetworkopen.2022.38507)
Supplement: Supplement. — eTable 1. Patient Characteristics Stratified by Race and COVID-19 Period eTable 2. Treatments and Organ Support Stratified by Race and COVID-19 Period eTable 3. Clinical Outcomes Stratified by Race and COVID-19 Period eTable 4. COVID-19 Treatments, Organ Support Measures, and Outcomes in a 1:1 Matched Cohort Stratified by Time Periods eTable 5. Comparison of Health Care–Associated Infection Rates, Number of Beds, Location, and Complexity Level of Hospitals in the 20th vs 80th Percentile for Within-Center Race Odd Ratios for Steroid Treatment eTable 6. Model Specifications and Coefficients for Steroid Treatment eTable 7. Model Specifications and Coefficients for Remdesivir Treatment eTable 8. Model Specifications and Coefficients for Immunomodulation Treatment eTable 9. Model Specifications and Coefficients for Intensive Care Unit Admission eTable 10. Model Specifications and Coefficients for Mechanical Ventilation eTable 11. Model Specifications and Coefficients or Noninvasive Ventilation eTable 12. Model Specifications and Coefficients for Prone Positioning eTable 13. Model Specifications and Coefficients for Hospital Death eTable 14. Model Specifications and Coefficients for 60-day Death eTable 15. Model Specifications and Coefficients for Hospital Readmissions eFigure 1. Directed Acyclic Graph (DAG) Illustrating Potential Mediators of the Associations of Race With Treatment Outcomes and Potential Confounders of the Mediators eFigure 2. Unadjusted Treatment Outcomes by Race and Month eFigure 3. Distribution of Within-Center Adjusted Odds Ratios for Steroid Treatment eFigure 4. Most Common Readmission Diagnoses Stratified by Race and Time Period eFigure 5. Balance Plot for Covariates Before and After Matching [file jamanetwopen-e2238507-s001.pdf]

Supplemental Online Content

Castro AD, Mayr FB, Talisa VB, et al. Variation in clinical treatment and outcomes by race among US veterans hospitalized with COVID-19. *JAMA Netw Open*. 2022;5(10):e2238507. doi:10.1001/jamanetworkopen.2022.38507

eTable 1. Patient Characteristics Stratified by Race and COVID-19 Period
eTable 2. Treatments and Organ Support Stratified by Race and COVID-19 Period
eTable 3. Clinical Outcomes Stratified by Race and COVID-19 Period
eTable 4. COVID-19 Treatments, Organ Support Measures, and Outcomes in a 1:1 Matched Cohort Stratified by Time Periods
eTable 5. Comparison of Health Care–Associated Infection Rates, Number of Beds, Location, and Complexity Level of Hospitals in the 20th vs 80th Percentile for Within-Center Race Odd Ratios for Steroid Treatment
eTable 6. Model Specifications and Coefficients for Steroid Treatment
eTable 7. Model Specifications and Coefficients for Remdesivir Treatment
eTable 8. Model Specifications and Coefficients for Immunomodulation Treatment
eTable 9. Model Specifications and Coefficients for Intensive Care Unit Admission
eTable 10. Model Specifications and Coefficients for Mechanical Ventilation
eTable 11. Model Specifications and Coefficients or Noninvasive Ventilation
eTable 12. Model Specifications and Coefficients for Prone Positioning
eTable 13. Model Specifications and Coefficients for Hospital Death
eTable 14. Model Specifications and Coefficients for 60-day Death
eTable 15. Model Specifications and Coefficients for Hospital Readmissions
eFigure 1. Directed Acyclic Graph (DAG) Illustrating Potential Mediators of the Associations of Race With Treatment Outcomes and Potential Confounders of the Mediators
eFigure 2. Unadjusted Treatment Outcomes by Race and Month
eFigure 3. Distribution of Within-Center Adjusted Odds Ratios for Steroid Treatment
eFigure 4. Most Common Readmission Diagnoses Stratified by Race and Time Period
eFigure 5. Balance Plot for Covariates Before and After Matching

This supplemental material has been provided by the authors to give readers additional information about their work.

© 2022 Castro AD et al. *JAMA Network Open.*

eTable 1. Patient Characteristics Stratified by Race and COVID-19 Period

	**Period 1 (N=1,652)**		**Period 2 (N=3,217)**		**Period 3 (N=17,409)**		**Period 4 (N=8,690)**		**Period 5 (N=12,254)**	
	Mar 1 to May 17, 2020		May 18 to Sep 1, 2020		Sep 2 2020, to Jun 7, 2021		Jun 8 to Oct 30, 2021		Oct 31 2021 to Feb 28, 2022	
Characteristic	White n=731 (44.2)	Black n=921 (55.8)	White n=1,946 (60.5)	Black n=1,271 (39.5)	White n=12,603 (72.4)	Black n=4,806 (27.6)	White n=6,645 (76.5)	Black n=2,045 (23.5)	White n=9,162 (74.8)	Black n=3,092 (25.2)
Age	73 (64, 80)	69 (61, 75)	72 (64, 78)	67 (58, 74)	73 (65, 79)	67 (59, 74)	72 (61, 76)	65 (56, 72)	72 (63, 78)	67 (60, 74)
Male sex	700 (95.8)	871 (94.6)	1,847 (94.9)	1,170 (92.1)	12,064 (95.7)	4,406 (91.7)	6,310 (95.0)	1,824 (89.2)	8,708 (95.0)	2,817 (91.1)
Ethnicity										
Hispanic	95 (13.0)	33 (3.6)	318 (16.3)	19 (1.5)	1,101 (8.7)	66 (1.4)	545 (8.2)	28 (1.4)	699 (7.6)	51 (1.6)
Non-Hispanic	621 (85.0)	873 (94.8)	1,585 (81.4)	1,224 (96.3)	11,041 (87.6)	4,578 (95.3)	5,812 (87.5)	1,943 (95.0)	8,142 (88.9)	2,919 (94.4)
Unknown	15 (2.0)	15 (1.6)	43 (2.2)	28 (2.2)	461 (3.7)	162 (3.4)	288 (4.3)	74 (3.6)	321 (3.5)	122 (3.9)
Area Deprivation Index	90 (78, 99)	94 (86, 108)	95 (87, 102)	97 (90, 105)	93 (85, 101)	96 (89, 104)	95 (87, 103)	97 (90, 104)	93 (85, 101)	94 (86, 104)
Vaccination status										
None	731 (100.0)	921 (100)	1,946 (100)	1,271 (100)	11,228 (89.1)	4,295 (89.4)	3,749 (56.4)	1,173 (57.4)	4,174 (45.6)	1,174 (38.0)
Partially					707 (5.6)	289 (6.0)	196 (2.9)	108 (5.3)	188 (2.1)	93 (3.0)
Fully					663 (5.3)	220 (4.6)	2,594 (39.0)	734 (35.9)	3,101 (33.8)	1,105 (35.7)
Boosted					5 (0.04)	2 (0.04)	106 (1.6)	30 (1.5)	1,699 (18.5)	720 (23.3)
Charlson Comorbidity Index	4 (2, 6)	4 (2, 6)	3 (2, 6)	4 (2, 6)	3 (1, 5)	3 (1, 5)	2 (1, 4)	2 (1, 4)	2 (1, 4)	3 (1, 5)
Coronary artery disease	274 (37.5)	228 (24.8)	698 (35.9)	301 (23.7)	4,631 (36.7)	1,230 (25.6)	2,176 (32.7)	481 (23.5)	3,455 (37.7)	900 (29.1)
Malignancy	145 (19.8)	215 (23.3)	377 (19.4)	267 (21.0)	2,823 (22.4)	1,097 (22.8)	1,456 (21.9)	391 (19.1)	2,184 (23.8)	792 (25.6)
Chronic kidney disease	218 (29.8)	307 (33.3)	486 (25.0)	387 (30.4)	3,326 (26.4)	1,571 (32.7)	1,549 (23.3)	589 (28.8)	2,555 (27.9)	1,176 (38.0)
Chronic obstructive lung disease	216 (29.5)	229 (24.9)	551 (28.3)	285 (22.4)	3,808 (30.2)	1,032 (21.5)	1,884 (28.3)	429 (21.0)	2,956 (32.3)	806 (26.1)
Diabetes	374 (51.2)	502 (54.5)	957 (49.2)	678 (53.3)	6,261 (49.7)	2,610 (54.3)	3,048 (45.o)	974 (47.6)	4,249 (46.4)	1,539 (49.8)
Hypertension	574 (78.5)	771 (83.7)	1,504 (77.3)	1,031 (81.1)	10,108 (80.2)	4,060 (84.5)	5,078 (76.4)	1,614 (78.9)	7,214 (78.7)	2,570 (83.1)

© 2022 Castro AD et al. *JAMA Network Open.*

eTable 2. Treatments and Organ Support Stratified by Race and COVID-19 Period
[1] denominator used was the number of patients who required any supplemental oxygen

	Period 1 (N=1,652)		Period 2 (N=3,217)		Period 3 (N=17,409)		Period 4 (N=8,690)		Period 5 (N=12,254)	
	Mar 1 to May 17, 2020		May 18 to Sep 1, 2020		Sep 2 to Jun 7, 2021		Jun 8 to Oct 30, 2021		Oct 31 to Feb 28, 2022	
Characteristic	White[1] n=731 (44.2)	Black[1] n=921 (55.8)	White[1] n=1,946 (60.5)	Black[1] n=1,271 (39.5)	White[1] n=12,603 (72.4)	Black[1] n=4,806 (27.6)	White[1] n=6,645 (76.5)	Black[1] n=2,045 (23.5)	White[1] n=9,162 (74.8)	Black[1] n=3,092 (25.2)
Any oxygen requirement	604 (82.6)	758 (82.3)	1,401 (72.0)	870 (68.5)	9,163 (72.7))	3,286 (68.4)	5,337 (80.3)	1,461 (71.4)	6,386 (69.7)	1,738 (56.2)
Monoclonal antibodies					101 (0.8)	43 (0.9)	374 (5.6)	105 (5.1)	447 (4.9)	125 (4.0)
Antivirals prior to admission									47 (0.5)	17 (0.5)
[1]Remdesivir	14 (2.3)	16 (2.1)	445 (31.8)	212 (24.4)	5,143 (56.1)	1,708 (52.0)	3,655 (68.5)	921 (63.0)	3,839 (60.1)	910 (52.4)
Day of first dose	5 (4, 6)	7 (3, 13)	2 (1, 3)	2 (1, 3)	1 (1, 2)	2 (1, 2)	1 (1, 2)	2 (1, 2)	1 (1, 2)	1 (1, 2)
[1]Systemic Steroids	55 (9.1)	64 (8.4)	844 (60.2)	498 (57.2)	6,470 (70.6)	2,240 (68.2))	4,248 (79.6)	1,081 (74.0)	4,505 (70.5)	1,017 (58.5)
Day of first dose	7 (2, 12)	4 (2, 9)	2 (1, 2)	2 (1, 3)	2 (1, 2)	2 (1, 2)	2 (1, 2)	2 (1, 2)	2 (1, 2)	2 (1, 2)
[1]Immunomodulators	51 (8.4)	67 (8.8)	74 (5.3)	35 (4.0)	227 (2.5)	98 (3.0)	1,035 (19.4)	208 (14.2	956 (15.0)	154 (8.9)
Day of first dose	5 (3, 7)	5 (3, 7)	7 (4, 9)	5 (2, 6)	3 (2, 6)	3 (2, 4)	3 (2, 4)	3 (2, 4)	2 (2, 4)	2 (2, 5)
ICU admission	333 (45.6)	423 (45.9)	946 (48.6)	543 (42.7)	5,299 (42.0)	1,888 (39.3)	3,148 (47.4)	829 (40.5)	3,701 (40.4)	1,123 (36.4)
Organ Support										
High flow oxygen	202 (27.6)	237 (25.7)	523 (26.9)	293 (23.1)	3,196 (25.4)	1,063 (22.1)	2,241 (33.7)	550 (26.9)	2,019 (22.0)	450 (14.6)
Non-invasive ventilation	144 (19.7)	171 (18.6)	460 (23.6)	238 (18.7)	3,018 (23.9)	986 (20.5)	1,962 (29.5)	441 (21.6)	1,966 (21.5)	493 (15.9)
Mechanical ventilation (MV)	128 (17.5)	204 (22.1)	223 (11.5)	121 (9.5)	984 (7.8)	378 (7.9)	738 (11.1)	201 (9.8)	524 (5.7)	153 (4.9)
Days of MV	5 (2, 13)	7 (3, 12)	6 (1, 12)	4 (1, 10)	4 (0, 11)	4 (0, 12)	5 (1, 12)	4 (1, 10)	3 (0, 9)	2 (0, 7)
Time from admission to MV	2 (0, 4)	2 (0, 4)	3 (1, 7)	4 (1, 7)	3 (1, 7)	4 (1, 10)	3 (1, 7)	4 (1, 9)	3 (1, 7)	4 (1, 9)
Prone Positioning	61 (47.7)	83 (40.7)	112 (50.2)	53 (43.8)	499 (50.7)	177 (47)	393 (53.3)	91 (45.3)	233 (44.5)	48 (31.4)
Extracorporeal Membrane Oxygenation	0 (0.0)	1 (0.1)	1 (0.0)	0 (0.0)	5 (0.0)	0 (0.0)	4 (0.1)	0 (0.0)	1 (0.0)	0 (0.0)
Vasopressor support	155 (21.2)	219 (23.8)	258 (13.3)	138 (10.9)	1,183 (9.8)	465 (9.7)	802 (12.1)	217 (10.6)	754 (8.2)	223 (7.2)
Renal Replacement Therapy	52 (7.1)	121 (13.1)	76 (3.9)	103 (8.2)	423 (3.5)	405 (8.8)	224 (3.4)	153 (7.5)	301 (3.3)	264 (8.5)

© 2022 Castro AD et al. *JAMA Network Open.*

eTable 3. Clinical Outcomes Stratified by Race and COVID-19 Period

	Period 1 (N=1,652)		Period 2 (N=3,217)		Period 3 (N=17,409		Period 4 (N=8,690)		Period 5 (N=12,254)	
	Mar 1 to May 17, 2020		May 18 to Sep 1, 2020		Sep 2 to Jun 7, 2021		Jun 8 to Oct 30, 2021		Oct 31 to Feb 28, 2022	
Characteristic	White[1] n=731 (44.2)	Black[1] n=921 (55.8)	White[1] n=1,946 (60.5)	Black[1] n=1,271 (39.5)	White[1] n=12,603 (72.4)	Black[1] n=4,806 (27.6)	White[1] n=6,645 (76.5)	Black[1] n=2,045 (23.5)	White[1] n=9,162 (74.8)	Black[1] n=3,092 (25.2)
ICU admission	333 (45.6)	423 (45.9)	946 (48.6)	543 (42.7)	5,299 (42.0)	1,888 (39.3)	3,148 (47.4)	829 (40.5)	3,701 (40.4)	1,123 (36.3)
ICU length of stay (LOS)	7 (3, 14)	7 (3, 12)	6 (2, 13)	6 (2, 12)	5 (2, 10)	5 (2, 10)	6 (2, 11)	5 (2, 10)	4 (2, 9)	4 (2, 8)
Hospital LOS	7 (3, 14)	7 (3, 12)	6 (3, 13)	6 (3, 11)	5 (2, 10)	5 (2, 10)	5 (3, 10)	5 (2, 10)	4 (2, 9)	4 (2, 9)
Hospital mortality	194 (26.5)	217 (23.6)	253 (13.0)	108 (8.5)	1,304 (10.3)	424 (8.8)	815 (12.3)	200 (9.8)	745 (8.1)	180 (5.8)
60-day Mortality	263 (36.0)	272 (29.5)	457 (23.5)	193 (15.2)	2,412 (19.1)	729 (15.2)	1,224 (18.4)	276 (13.5)	1,339 (14.6)	348 (11.3)
30-day Readmission	83 (15.5)	98 (13.9)	264 (13.6)	154 (12.1)	1,682 (13.3)	620 (12.9)	807 (12.1)	243 (11.9)	1,271 (13.9)	446 (14.4)

© 2022 Castro AD et al. *JAMA Network Open.*

eTable 4. COVID-19 Treatments, Organ Support Measures, and Outcomes in a 1:1 Matched Cohort Stratified by Time Periods[1]

	**Periods 1-2 (N=468)** Mar 1 to Sep 1, 2020			**Periods 3-5 (N=3,594)** Sep 2 2020 to Feb 28, 2022			**All Periods (N=4,062)** Mar 1, 2020, to Feb 28, 2022		
	White (n=234)	Black (n=234)	P value	White (n=1,797)	Black (n=1,797)	P value	White (n=2,031)	Black (n=2,031)	P value
**Demographics**									
Age	73 (69, 78)	72 (68, 78)	0.53	72 (65, 76)	72 (65, 76)	0.15	72 (66, 77)	72 (65, 76)	0.12
Charlson Index	5 (3, 7)	5 (3, 6)	0.91	3 (1, 5)	3 (1, 6)	0.23	4 (2, 5)	4 (2, 6)	0.25
ADI	95 (91, 102)	97 (92, 102)	0.43	96 (90, 102)	96 (91, 103)	0.23	96 (90, 102)	96 (91, 103)	0.21
Male gender	232 (99.1)	232 (99.1)	1.00	1,764 (98.2)	1,764 (98.2)	1.00	2,457 (98.6)	2,457 (98.6)	1.00
**COVID-19 Treatments**									
Systemic Steroids	86 (36.4)	95 (40.6)	0.39	1,265 (70.4)	1,193 (66.4)	0.01	1,351 (66.5)	1,288 (63.4)	0.04
Remdesivir	46 (19.7)	31 (13.2)	0.06	1,061 (59.0)	971 (54.0)	0.002	1,107 (54.5)	1,002 (49.3)	<0.001
Immunomodulators	12 (5.1)	9 (3.8)	0.50	90 (5.0)	83 (4.6)	0.59	102 (5.0)	92 (4.5)	0.46
**Organ support measures**									
ICU admission	117 (50.0)	99 (42.3)	0.10	758 (42.2)	757 (42.1)	0.97	875 (43.1)	856 (42.1)	0.55
Low-flow oxygen	228 (97.4)	231 (98.7)	0.50	1,754 (97.6)	1,746 (97.2)	0.40	1,982 (97.6)	1,977 (97.3)	0.62
High-flow oxygen	80 (34,2)	62 (26.5)	0.07	529 (29.4)	499 (27.8)	0.27	609 (30.0)	561 (27.6)	0.10
Non-invasive ventilation	42 (17.9)	42 (17.9)	1.00	357 (19.9)	357 (19.9)	1.00	399 (19.6)	399 (19.6)	1.00
Mechanical ventilation (MV)	23 (9.8)	23 (9.8)	1.00	75 (4.2)	75 (4.2)	1.00	98 (4.8)	98 (4.8)	1.00
Ventilator days	5 (3, 10)	4 (1, 10)	0.27	3 (0, 9)	4 (0, 12)	0.42	4 (0, 9)	4 (0, 12)	0.72
Time to initation of MV	5 (1, 10)	4 (1, 12)	0.90	4 (1, 10)	6 (2, 15)	0.22	4 (1, 10)	6 (1, 14)	0.25
Prone Positioning	15 (65.2)	11 (47.8)	0.37	38 (50.7)	47 (62.7)	0.19	53 (54.1)	58 (59.2)	0.58
**Outcomes**									
Hospital Mortality	46 (19.7)	37 (15.8)	0.28	161 (9.0)	160 (8.9)	0.95	207 (9.9)	197 (9.7)	0.60
60-day Mortality	80 (34.2)	60 (25.5)	0.04	328 (18.3)	316 (17.6)	0.60	408 (20.1)	376 (18.5)	0.20
30-day Readmission	37 (19.7)	41 (20.8)	0.62	290 (17.7)	307 (18.8)	0.45	327 (17.9)	348 (19.0)	0.56

[1]We matched Black and White subjects with a documented need for supplemental oxygen using coarsened exact matching on 10-year age intervals, sex, ethnicity, Charlson Index categories (0,1,2 or >2), area deprivation index categories, invasive and non-invasive mechanical ventilation, vaccination status, admission month, location, and preexisting chronic kidney and liver disease.

© 2022 Castro AD et al. *JAMA Network Open.*

eTable 5. Comparison of Health Care–Associated Infection Rates, Number of Beds, Location, and Complexity Level of Hospitals in the 20th vs 80th Percentile for Within-Center Race Odd Ratios for Steroid Treatment Data presented as means with standard errors of means. The odds ratio reflects the odds of receiving steroids for COVID-19 in Black vs. White patients. An odds ratio of less than 1 indicates a lower likelihood of Black patients receiving steroids than White patients.

	[1]CLABSI	[2]CAUTI	Beds	Location	[3]Complexity level
**20th percentile**	1.70 (0.27)	0.88 (0.15)	94 (9)	2/21 rural	17 high, 3 medium, 1 low
**80th percentile**	1.41 (0.21)	0.65 (0.10)	118 (13)	0/24 rural	22 high, 1 medium, 1 low

[1]Number of central line-associated bloodstream infections (CLABSI) per 1000 device days
[2]Number of catheter-associated urinary tract infections (CAUTI) per 1000 device days
[3]VA medical centers are categorized into high-complexity, medium complexity, and low complexity facilities. High-complexity facilities typically serve medium to high-risk patients and harbor complex clinical and medium to large-sized research and teaching programs. Medium and low complexity facilities serve low-risk patients and harbor few or no complex clinical and research/teaching programs

© 2022 Castro AD et al. *JAMA Network Open.*

eTable 6. Model Specifications and Coefficients for Steroid Treatment

Characteristic	beta	95% CI[1]	p-value
(Intercept)	-3.6	-4.1, -3.0	<0.001
ns(Age, df = 5)1	0.47	0.17, 0.78	0.002
ns(Age, df = 5)2	0.73	0.38, 1.1	<0.001
ns(Age, df = 5)3	0.30	0.07, 0.53	0.010
ns(Age, df = 5)4	1.0	0.27, 1.8	0.008
ns(Age, df = 5)5	-0.20	-0.58, 0.18	0.307
Male sex	0.00	-0.12, 0.12	0.983
Charlson Index	-0.04	-0.05, -0.03	<0.001
Chronic kidney disease	-0.12	-0.19, -0.05	<0.001
Chronic liver disease	-0.30	-0.39, -0.21	<0.001
Black race	-0.13	-0.22, -0.04	0.004
Proportion of Black patients	-0.40	-0.73, -0.07	0.018
Non-Hispanic Ethnicity	-0.15	-0.27, -0.03	0.012
Unknown Ethnicity	-0.16	-0.34, 0.02	0.090
Area deprivation index	0.00	0.00, 0.00	0.001
ICU admission	0.39	0.33, 0.44	<0.001
Mechanical ventilatoin	1.1	1.0, 1.3	<0.001
Vasopressors	0.16	0.05, 0.27	0.005
Renal replacement therapy	-0.32	-0.45, -0.20	<0.001
Number of mRNA vaccinations			
1	-0.23	-0.38, -0.09	0.001
2	-0.71	-0.79, -0.63	<0.001
3	-1.0	-1.1, -0.88	<0.001
[2]Month of hospital admission			
1	0.00	—	
2	0.38	-0.06, 0.81	0.089
3	0.08	-0.47, 0.63	0.778
4	2.5	2.1, 2.9	<0.001
5	3.8	3.4, 4.2	<0.001
6	3.7	3.3, 4.1	<0.001
12	3.5	3.2, 3.9	<0.001
24	3.6	3.2, 4.0	<0.001
Time between positive test and admission	0.03	0.02, 0.04	<0.001
**Random effects**			
Sta3n.sd__(Intercept)	0.24		
Sta3n.1.sd__test_to_hosp	0.04		
Sta3n.2.sd__race_cat2	0.26		

[1]CI = Confidence Interval

No. Obs. = 31,004; Sigma = 1.00; Log-likelihood = -16,746; AIC = 33,590; BIC = 33,998; Deviance = 33,087; Residual df = 30,955

[2]Months 7 to 11 and 13 to 23 omitted from table due to space constraints

© 2022 Castro AD et al. *JAMA Network Open.*

eTable 7. Model Specifications and Coefficients for Remdesivir Treatment

Characteristic	beta	95% CI[1]	p-value
(Intercept)	-17	-223, 190	0.875
ns(Age, df = 5)1	0.44	0.16, 0.72	0.002
ns(Age, df = 5)2	0.68	0.35, 1.0	<0.001
ns(Age, df = 5)3	0.23	0.01, 0.44	0.041
ns(Age, df = 5)4	0.82	0.12, 1.5	0.022
ns(Age, df = 5)5	-0.31	-0.68, 0.06	0.100
Male sex	-0.10	-0.21, 0.02	0.092
Charlson Index	-0.04	-0.05, -0.03	<0.001
Chronic kidney disease	-0.38	-0.45, -0.32	<0.001
Chronic liver disease	-0.20	-0.28, -0.12	<0.001
Black race	-0.12	-0.18, -0.05	<0.001
Proportion of Black patients	-0.38	-0.75, -0.01	0.043
Non-Hispanic Ethnicity	-0.15	-0.25, -0.04	0.008
Unknown Ethnicity	-0.20	-0.37, -0.04	0.018
Area deprivation index	0.00	0.00, 0.00	0.329
ICU admission	0.23	0.17, 0.28	<0.001
Mechanical ventilation	0.57	0.46, 0.68	<0.001
Vasopressors	-0.06	-0.16, 0.04	0.241
Renal replacement therapy	-1.0	-1.1, -0.90	<0.001
Number of mRNA vaccinations			
1	-0.07	-0.21, 0.07	0.318
2	-0.45	-0.52, -0.37	<0.001
3	-0.55	-0.68, -0.43	<0.001
Month of hospital admission			
1	—	—	
2	12	-195, 219	0.910
3	15	-192, 221	0.889
4	16	-191, 222	0.882
5	16	-191, 222	0.882
6	16	-191, 222	0.882
12	17	-190, 223	0.874
24	17	-190, 224	0.873
Time between positive test and admission	-0.01	-0.02, 0.00	0.050
**Random effects**			
Sta3n.sd__(Intercept)	0.30		
Sta3n.1.sd__test_to_hosp	0.03		
Sta3n.2.sd__race_cat2	0.11		

[1]CI = Confidence Interval

No. Obs. = 31,004; Sigma = 1.00; Log-likelihood = -18,688; AIC = 37,474; BIC = 37,883; Deviance = 36,980; Residual df = 30,955[2]Months 7 to 11 and 13 to 23 omitted from table due to space constraints

© 2022 Castro AD et al. *JAMA Network Open.*

eTable 8. Model Specifications and Coefficients for Immunomodulation Treatment

Characteristic	beta	95% CI[1]	p-value
(Intercept)	-4.3	-5.0, -3.5	<0.001
ns(Age, df = 5)1	0.49	0.01, 1.0	0.047
ns(Age, df = 5)2	0.48	-0.08, 1.0	0.094
ns(Age, df = 5)3	0.23	-0.19, 0.65	0.280
ns(Age, df = 5)4	0.57	-0.67, 1.8	0.368
ns(Age, df = 5)5	-0.92	-1.8, -0.05	0.039
Male sex	0.19	-0.01, 0.40	0.061
Charlson Index	-0.08	-0.10, -0.05	<0.001
Chronic kidney disease	-0.07	-0.19, 0.06	0.295
Chronic liver disease	-0.18	-0.33, -0.02	0.028
Black race	-0.27	-0.39, -0.14	<0.001
Proportion of Black patients	-0.03	-0.48, 0.43	0.905
Non-Hispanic Ethnicity	-0.21	-0.39, -0.03	0.021
Unknown Ethnicity	-0.16	-0.44, 0.12	0.260
Area deprivation index	0.00	-0.01, 0.00	0.032
ICU admission	1.9	1.7, 2.0	<0.001
Mechanical ventilation	1.2	1.1, 1.3	<0.001
Vasopressors	0.17	0.03, 0.31	0.016
Renal replacement therapy	-0.33	-0.51, -0.14	<0.001
Number of mRNA vaccinations			
1	-0.37	-0.63, -0.10	0.007
2	-0.70	-0.82, -0.58	<0.001
3	-1.1	-1.3, -0.83	<0.001
Month of hospital admission			
1	—	—	
2	0.57	0.13, 1.0	0.012
3	-0.08	-0.68, 0.53	0.805
4	-0.04	-0.57, 0.49	0.877
5	0.02	-0.43, 0.48	0.917
6	-1.5	-2.3, -0.77	<0.001
12	-0.34	-0.84, 0.16	0.184
24	0.82	0.35, 1.3	<0.001
Time between positive test and admission	0.02	0.00, 0.03	0.035
**Random effects**			
Sta3n.sd__(Intercept)	0.30		
Sta3n.1.sd__test_to_hosp	0.00		
Sta3n.2.sd__race_cat2	0.20		

[1]CI = Confidence Interval

No. Obs. = 31,004; Sigma = 1.00; Log-likelihood = -6,928; AIC = 13,955; BIC = 14,364; Deviance = 13,670; Residual df = 30,955

[2]Months 7 to 11 and 13 to 23 omitted from table due to space constraints

© 2022 Castro AD et al. *JAMA Network Open.*

eTable 9. Model Specifications and Coefficients for Intensive Care Unit Admission

Characteristic	beta	95% CI[1]	p-value
(Intercept)	-1.7	-2.1, -1.3	<0.001
ns(Age, df = 5)1	0.07	-0.16, 0.30	0.542
ns(Age, df = 5)2	0.19	-0.07, 0.45	0.160
ns(Age, df = 5)3	0.23	0.04, 0.41	0.018
ns(Age, df = 5)4	0.09	-0.49, 0.66	0.769
ns(Age, df = 5)5	0.16	-0.16, 0.47	0.335
Male sex	0.01	-0.09, 0.11	0.870
Charlson Index	0.03	0.02, 0.04	<0.001
Black race	-0.05	-0.13, 0.02	0.165
Proportion of Black patients	-0.17	-0.74, 0.40	0.555
Non-Hispanic Ethnicity	0.04	-0.06, 0.14	0.490
Unknown Ethnicity	0.04	-0.11, 0.20	0.572
Area deprivation index	0.00	0.00, 0.01	<0.001
ICU admission	1.9	1.7, 2.0	<0.001
Mechanical ventilation	17	-143, 178	0.831
Non-invasive ventilation	0.88	0.82, 0.93	<0.001
Vasopressors	1.2	1.1, 1.4	<0.001
Renal replacement therapy	0.46	0.33, 0.58	<0.001
Number of mRNA vaccinations			
1	-0.16	-0.28, -0.04	0.012
2	-0.13	-0.20, -0.06	<0.001
3	-0.09	-0.20, 0.02	0.093
Month of hospital admission			
1	—	—	
2	0.07	-0.22, 0.36	0.617
3	0.37	0.05, 0.69	0.024
4	0.46	0.16, 0.75	0.002
5	0.25	-0.02, 0.52	0.073
6	0.30	0.02, 0.58	0.038
12	0.06	-0.21, 0.33	0.650
24	-0.22	-0.49, 0.05	0.111
Time between positive test and admission	-0.01	-0.02, 0.00	0.002
Steroid treatment	0.37	0.31, 0.43	<0.001
Remdesivir treatment	0.06	0.00, 0.12	0.035
Immunomodulatory treatment	1.7	1.6, 1.8	<0.001
**Random effects**			
Sta3n.sd__(Intercept)	0.53		
Sta3n.1.sd__test_to_hosp	0.00		
Sta3n.2.sd__race_cat2	0.22		

[1]CI = Confidence Interval

No. Obs. = 43,222; Sigma = 1.00; Log-likelihood = -23,006; AIC = 46,112; BIC = 46,545; Deviance = 45,484; Residual df = 43,172

[2]Months 7 to 11 and 13 to 23 omitted from table due to space constraints

© 2022 Castro AD et al. *JAMA Network Open.*

eTable 10. Model Specifications and Coefficients for Mechanical Ventilation

Characteristic	beta	95% CI[1]	p-value
(Intercept)	-4.8	-5.8, -3.9	<0.001
ns(Age, df = 5)1	0.73	0.03, 1.4	0.041
ns(Age, df = 5)2	0.67	-0.12, 1.5	0.097
ns(Age, df = 5)3	0.36	-0.15, 0.86	0.169
ns(Age, df = 5)4	-0.26	-2.0, 1.5	0.772
ns(Age, df = 5)5	-1.1	-2.1, -0.11	0.030
Male sex	0.09	-0.16, 0.33	0.483
Charlson Index	-0.04	-0.06, -0.02	<0.001
Black race	0.09	-0.04, 0.22	0.159
Proportion of Black patients	0.49	0.02, 1.0	0.040
Non-Hispanic Ethnicity	0.19	-0.01, 0.39	0.061
Unknown Ethnicity	-0.04	-0.38, 0.30	0.821
Area deprivation index	0.01	0.00, 0.01	<0.001
Low flow oxygen supplementation	0.42	0.24, 0.60	<0.001
Non-invasive ventilation	1.3	1.2, 1.4	<0.001
Vasopressors	3.6	3.5, 3.7	<0.001
Renal replacement therapy	0.43	0.27, 0.59	<0.001
Number of mRNA vaccinations			
1	-0.15	-0.44, 0.15	0.326
2	-0.26	-0.43, -0.10	0.002
3	-0.21	-0.52, 0.09	0.171
Month of hospital admission			
1	—	—	
2	-0.73	-1.1, -0.33	<0.001
3	-1.1	-1.6, -0.56	<0.001
4	-1.7	-2.1, -1.2	<0.001
5	-1.6	-2.0, -1.2	<0.001
6	-1.8	-2.2, -1.4	<0.001
12	-2.3	-2.7, -1.9	<0.001
24	-2.6	-3.1, -2.1	<0.001
Time between positive test and admission	0.01	0.00, 0.03	0.092
Steroid treatment	1.1	0.91, 1.2	<0.001
Remdesivir treatment	0.16	0.04, 0.28	0.009
Immunomodulatory treatment	1.3	1.2, 1.5	<0.001
**Random effects**			
Sta3n.sd__(Intercept)	0.29		
Sta3n.1.sd__test_to_hosp	0.00		
Sta3n.2.sd__race_cat2	0.10		

[1]CI = Confidence Interval

No. Obs. = 43,222; Sigma = 1.00; Log-likelihood = -6,099; AIC = 12,299; BIC = 12,732; Deviance = 12,055; Residual df = 43,172

[2]Months 7 to 11 and 13 to 23 omitted from table due to space constraints

© 2022 Castro AD et al. *JAMA Network Open.*

eTable 11. Model Specifications and Coefficients or Noninvasive Ventilation

Characteristic	beta	95% CI[1]	p-value
(Intercept)	-4.9	-5.3, -4.4	<0.001
ns(Age, df = 5)1	1.1	0.80, 1.5	<0.001
ns(Age, df = 5)2	1.3	1.0, 1.7	<0.001
ns(Age, df = 5)3	-0.04	-0.29, 0.20	0.718
ns(Age, df = 5)4	2.2	1.4, 3.0	<0.001
ns(Age, df = 5)5	-0.93	-1.4, -0.49	<0.001
Male sex	0.37	0.25, 0.49	<0.001
Charlson Index	0.05	0.04, 0.06	<0.001
Black race	-0.20	-0.27, -0.12	<0.001
Proportion of Black patients	-0.32	-0.62, -0.02	0.035
Non-Hispanic Ethnicity	-0.05	-0.15, 0.06	0.402
Unknown Ethnicity	0.03	-0.13, 0.20	0.700
Area deprivation index	0.00	0.00, 0.00	0.046
Low flow oxygen supplementation	1.2	1.1, 1.3	<0.001
Vasopressors	1.4	1.3, 1.4	<0.001
Renal replacement therapy	0.05	-0.07, 0.16	0.420
Number of mRNA vaccinations			
1	0.00	-0.14, 0.14	0.986
2	0.10	0.02, 0.17	0.015
3	0.31	0.19, 0.44	<0.001
Month of hospital admission			
1	—	—	
2	0.05	-0.25, 0.35	0.739
3	0.20	-0.15, 0.54	0.272
4	0.34	0.03, 0.65	0.031
5	0.32	0.04, 0.60	0.025
6	0.28	-0.02, 0.58	0.065
12	0.45	0.17, 0.72	0.001
24	0.13	-0.15, 0.42	0.348
Time between positive test and admission	0.01	0.00, 0.01	0.163
Steroid treatment	0.39	0.32, 0.46	<0.001
Remdesivir treatment	0.19	0.13, 0.26	<0.001
Immunomodulatory treatment	1.2	1.2, 1.3	<0.001
**Random effects**			
Sta3n.sd__(Intercept)	0.22		
Sta3n.1.sd__test_to_hosp	0.00		
Sta3n.2.sd__race_cat2	0.19		

[1]CI = Confidence Interval

No. Obs. = 43,222; Sigma = 1.00; Log-likelihood = -19,447; AIC = 38,991; BIC = 39,416; Deviance = 38,625; Residual df = 43,173

[2]Months 7 to 11 and 13 to 23 omitted from table due to space constraints

© 2022 Castro AD et al. *JAMA Network Open.*

eTable 12. Model Specifications and Coefficients for Prone Positioning

Characteristic	beta	95% CI[1]	p-value
(Intercept)	-0.89	-2.1, 0.28	0.136
ns(Age, df = 5)1	-0.12	-0.92, 0.68	0.768
ns(Age, df = 5)2	0.01	-0.89, 0.91	0.988
ns(Age, df = 5)3	-0.90	-1.6, -0.22	0.009
ns(Age, df = 5)4	-1.0	-3.0, 1.0	0.324
ns(Age, df = 5)5	-2.5	-3.8, -1.1	<0.001
Male sex	-0.01	-0.39, 0.37	0.959
Charlson Index	-0.10	-0.13, -0.07	<0.001
Black race	0.00	-0.19, 0.20	0.981
Proportion of Black patients	-1.4	-2.1, -0.68	<0.001
Non-Hispanic Ethnicity	-0.22	-0.51, 0.08	0.153
Unknown Ethnicity	-0.35	-0.89, 0.18	0.197
Area deprivation index	0.00	-0.01, 0.00	0.170
Vasopressors	1.3	1.1, 1.5	<0.001
Renal replacement therapy	0.36	0.16, 0.56	<0.001
Number of mRNA vaccinations			
1	-0.45	-1.0, 0.06	0.085
2	-0.26	-0.54, 0.02	0.066
3	-0.44	-1.1, 0.19	0.174
Month of hospital admission			
1	—	—	
2	0.65	0.13, 1.2	0.014
3	0.19	-0.55, 0.93	0.617
4	0.23	-0.42, 0.89	0.479
5	-0.15	-0.72, 0.42	0.605
6	-0.23	-0.89, 0.42	0.485
12	-0.90	-1.6, -0.20	0.012
24	-1.6	-2.5, -0.67	<0.001
Time between positive test and admission	0.02	-0.01, 0.05	0.143
Steroid treatment	0.72	0.44, 1.0	<0.001
Remdesivir treatment	0.35	0.17, 0.54	<0.001
Immunomodulatory treatment	1.0	0.80, 1.2	<0.001
**Random effects**			
Sta3n.sd__(Intercept)	0.41		
Sta3n.1.sd__test_to_hosp	0.03		
Sta3n.2.sd__race_cat2	0.00		

[1]CI = Confidence Interval

No. Obs. = 3,654; Sigma = 1.00; Log-likelihood = -2,047; AIC = 4,191; BIC = 4,488; Deviance = 3,962; Residual df = 3,606

[2]Months 7 to 11 and 13 to 23 omitted from table due to space constraints

© 2022 Castro AD et al. *JAMA Network Open.*

eTable 13. Model Specifications and Coefficients for Hospital Death

Characteristic	beta	95% CI[1]	p-value
(Intercept)	-6.3	-7.5, -5.1	<0.001
ns(Age, df = 5)1	2.5	1.5, 3.6	<0.001
ns(Age, df = 5)2	2.7	1.6, 3.8	<0.001
ns(Age, df = 5)3	3.8	3.2, 4.4	<0.001
ns(Age, df = 5)4	5.4	3.0, 7.7	<0.001
ns(Age, df = 5)5	4.8	4.2, 5.5	<0.001
Male sex	0.33	0.08, 0.59	0.011
Charlson Index	0.07	0.05, 0.08	<0.001
Black race	-0.02	-0.15, 0.10	0.708
Proportion of Black patients	-0.16	-0.56, 0.23	0.421
Non-Hispanic Ethnicity	-0.02	-0.20, 0.16	0.814
Unknown Ethnicity	-0.39	-0.70, -0.09	0.011
Area deprivation index	0.00	0.00, 0.00	0.286
Mechanical ventilation	2.3	2.2, 2.4	<0.001
Vasopressors	1.4	1.3, 1.5	<0.001
Renal replacement therapy	0.60	0.45, 0.76	<0.001
Number of mRNA vaccinations			
1	-0.42	-0.68, -0.16	0.002
2	-0.56	-0.69, -0.43	<0.001
3	-0.68	-0.93, -0.43	<0.001
Month of hospital admission			
1	—	—	
2	0.09	-0.27, 0.45	0.620
3	-1.1	-1.6, -0.60	<0.001
4	-1.4	-1.8, -1.0	<0.001
5	-1.4	-1.7, -1.0	<0.001
6	-1.7	-2.1, -1.3	<0.001
12	-1.6	-2.0, -1.2	<0.001
24	-1.7	-2.1, -1.2	<0.001
Time between positive test and admission	0.00	-0.02, 0.01	0.557
Intensive care unit admission	1.0	0.89, 1.1	<0.001
Hospital length of stay	-0.02	-0.03, -0.02	<0.001
Steroid treatment	1.2	1.1, 1.3	<0.001
Remdesivir treatment	-0.07	-0.17, 0.03	0.165
Immunomodulatory treatment	1.1	0.93, 1.2	<0.001
**Random effects**			
Sta3n.sd__(Intercept)	0.23		
Sta3n.1.sd__test_to_hosp	0.00		
Sta3n.2.sd__race_cat2	0.23		

[1]CI = Confidence Interval

No. Obs. = 43,222; Sigma = 1.00; Log-likelihood = -8,219; AIC = 16,540; BIC = 16,982; Deviance = 16,273; Residual df = 43,171

[2]Months 7 to 11 and 13 to 23 omitted from table due to space constraints

© 2022 Castro AD et al. *JAMA Network Open.*

eTable 14. Model Specifications and Coefficients for 60-day Death

Characteristic	beta	95% CI[1]	p-value
(Intercept)	-5.3	-6.1, -4.4	<0.001
ns(Age, df = 5)1	1.9	1.2, 2.6	<0.001
ns(Age, df = 5)2	2.0	1.3, 2.8	<0.001
ns(Age, df = 5)3	3.2	2.8, 3.6	<0.001
ns(Age, df = 5)4	4.3	2.7, 5.9	<0.001
ns(Age, df = 5)5	4.7	4.3, 5.2	<0.001
Male sex	0.46	0.26, 0.66	<0.001
Charlson Index	0.12	0.11, 0.13	<0.001
Black race	-0.16	-0.25, -0.07	<0.001
Proportion of Black patients	-0.15	-0.46, 0.15	0.320
Non-Hispanic Ethnicity	0.11	-0.03, 0.25	0.114
Unknown Ethnicity	-0.23	-0.46, -0.01	0.045
Area deprivation index	0.00	0.00, 0.00	0.251
Mechanical ventilation	1.5	1.4, 1.6	<0.001
Vasopressors	1.5	1.4, 1.6	<0.001
Renal replacement therapy	0.49	0.36, 0.62	<0.001
Number of mRNA vaccinations			
1	-0.57	-0.76, -0.38	<0.001
2	-0.51	-0.61, -0.41	<0.001
3	-0.47	-0.63, -0.31	<0.001
Month of hospital admission			
1	-0.57	-0.76, -0.38	<0.001
2	-0.51	-0.61, -0.41	<0.001
3	-0.47	-0.63, -0.31	<0.001
4	-0.57	-0.76, -0.38	<0.001
5	-0.51	-0.61, -0.41	<0.001
6	-0.47	-0.63, -0.31	<0.001
12	-0.64	-1.0, -0.32	<0.001
24	-0.55	-0.87, -0.23	<0.001
Time between positive test and admission	-0.02	-0.03, -0.01	<0.001
Intensive care unit admission	0.86	0.79, 0.93	<0.001
Hospital length of stay	-0.01	-0.01, -0.01	<0.001
Steroid treatment	0.53	0.45, 0.61	<0.001
Remdesivir treatment	-0.25	-0.33, -0.17	<0.001
Immunomodulatory treatment	0.81	0.69, 0.92	<0.001
**Random effects**			
Sta3n.sd__(Intercept)	0.18		
Sta3n.1.sd__test_to_hosp	0.00		
Sta3n.2.sd__race_cat2	0.18		

[1]CI = Confidence Interval

No. Obs. = 43,222; Sigma = 1.00; Log-likelihood = -13,595; AIC = 27,291; BIC = 27,733; Deviance = 27,014; Residual df = 43,171

[2]Months 7 to 11 and 13 to 23 omitted from table due to space constraints

© 2022 Castro AD et al. *JAMA Network Open.*

eTable 15. Model Specifications and Coefficients for Hospital Readmissions

Characteristic	beta	95% CI[1]	p-value
(Intercept)	-3.2	-3.8, -2.6	<0.001
ns(Age, df = 5)1	0.44	0.06, 0.81	0.022
ns(Age, df = 5)2	0.49	0.06, 0.91	0.024
ns(Age, df = 5)3	0.71	0.44, 1.0	<0.001
ns(Age, df = 5)4	0.69	-0.22, 1.6	0.139
ns(Age, df = 5)5	0.81	0.39, 1.2	<0.001
Male sex	0.11	-0.03, 0.26	0.130
Charlson Index	0.13	0.12, 0.14	<0.001
Black race	-0.05	-0.13, 0.04	0.278
Proportion of Black patients	0.13	-0.14, 0.41	0.330
Non-Hispanic Ethnicity	-0.02	-0.15, 0.11	0.767
Unknown Ethnicity	0.08	-0.11, 0.28	0.411
Area deprivation index	0.00	0.00, 0.00	0.041
Mechanical ventilation	-0.88	-1.1, -0.67	<0.001
Vasopressors	1.1	1.0, 1.3	<0.001
Renal replacement therapy	0.35	0.22, 0.49	<0.001
Number of mRNA vaccinations			
1	0.12	-0.04, 0.28	0.142
2	0.00	-0.09, 0.09	0.975
3	0.11	-0.03, 0.24	0.116
Month of hospital admission			
1	—	—	
2	0.30	-0.12, 0.73	0.159
3	0.64	0.20, 1.1	0.004
4	0.52	0.10, 0.93	0.015
5	0.37	-0.02, 0.77	0.065
6	0.33	-0.08, 0.75	0.111
12	0.22	-0.17, 0.61	0.275
24	0.56	0.18, 1.0	0.004
Time between positive test and admission	-0.02	-0.03, -0.01	<0.001
Intensive care unit admission	0.85	0.78, 0.91	<0.001
Hospital length of stay	-0.03	-0.03, -0.02	<0.001
Steroid treatment	-0.02	-0.09, 0.06	0.663
Remdesivir treatment	-0.21	-0.29, -0.13	<0.001
Immunomodulatory treatment	-0.38	-0.56, -0.21	<0.001
**Random effects**			
Sta3n.sd__(Intercept)	0.15		
Sta3n.1.sd__test_to_hosp	0.00		
Sta3n.2.sd__race_cat2	0.16		

[1]CI = Confidence Interval

No. Obs. = 38,782; Sigma = 1.00; Log-likelihood = -14,687; AIC = 29,476; BIC = 29,913; Deviance = 29,224; Residual df = 38,731

[2]Months 7 to 11 and 13 to 23 omitted from table due to space constraints

© 2022 Castro AD et al. *JAMA Network Open.*

eFigure 1. Directed Acyclic Graph (DAG) Illustrating Potential Mediators of the Associations of Race With Treatment Outcomes and Potential Confounders of the Mediators

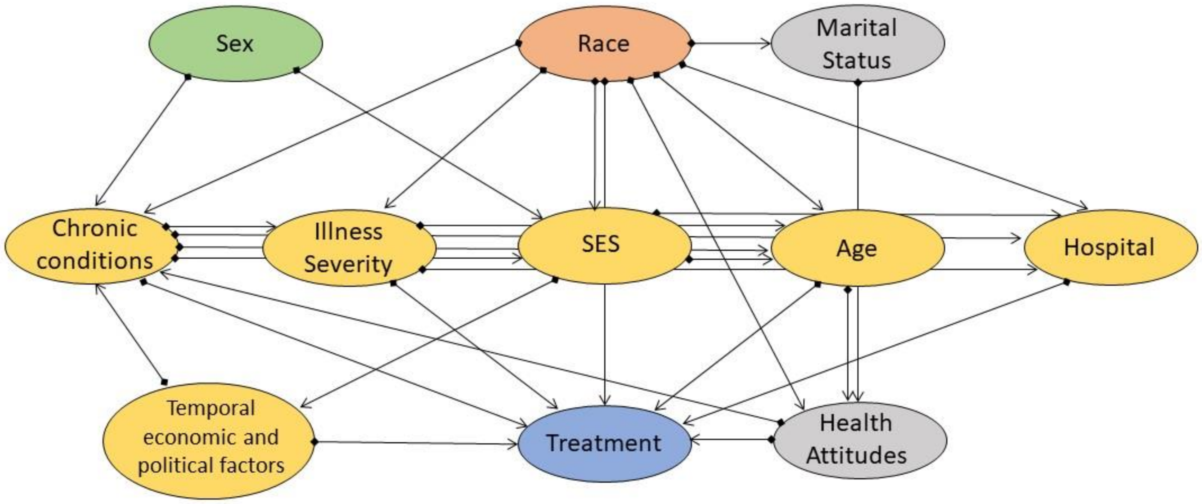

Color code:
Red: exposure
Yellow: mediator
Green: confounder
Blue: outcome
Gray: unobserved

© 2022 Castro AD et al. *JAMA Network Open.*

eFigure 2. Unadjusted Treatment Outcomes by Race and Month

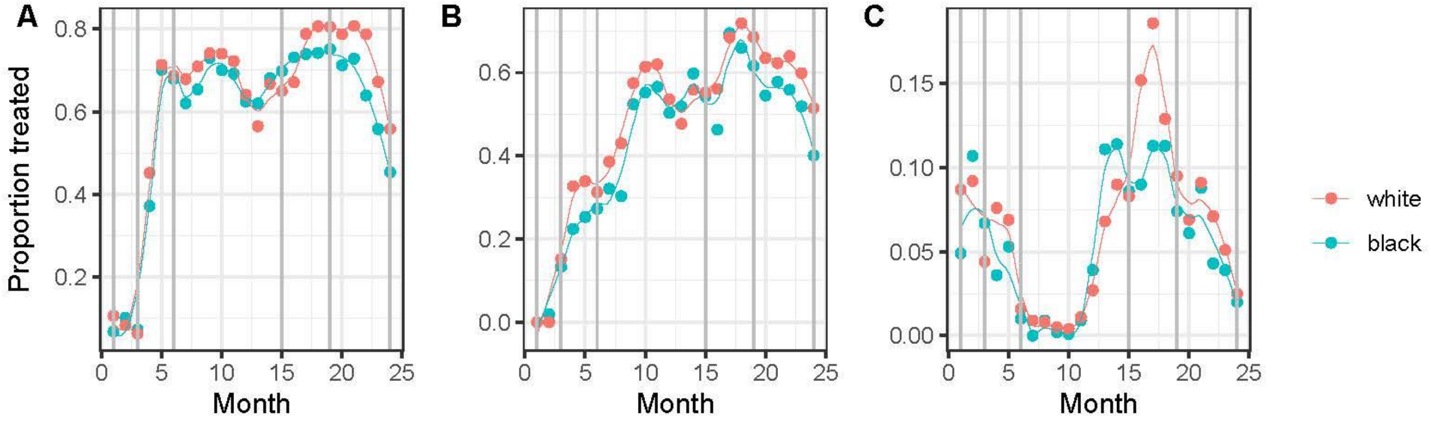

Proportion of patients who received treatment with steroids (A), Remdesivir (B), and immunomodulatory agents (C) by month of hospitalization**.**

© 2022 Castro AD et al. *JAMA Network Open.*

eFigure 3. Distribution of Within-Center Adjusted Odds Ratios for Steroid Treatment

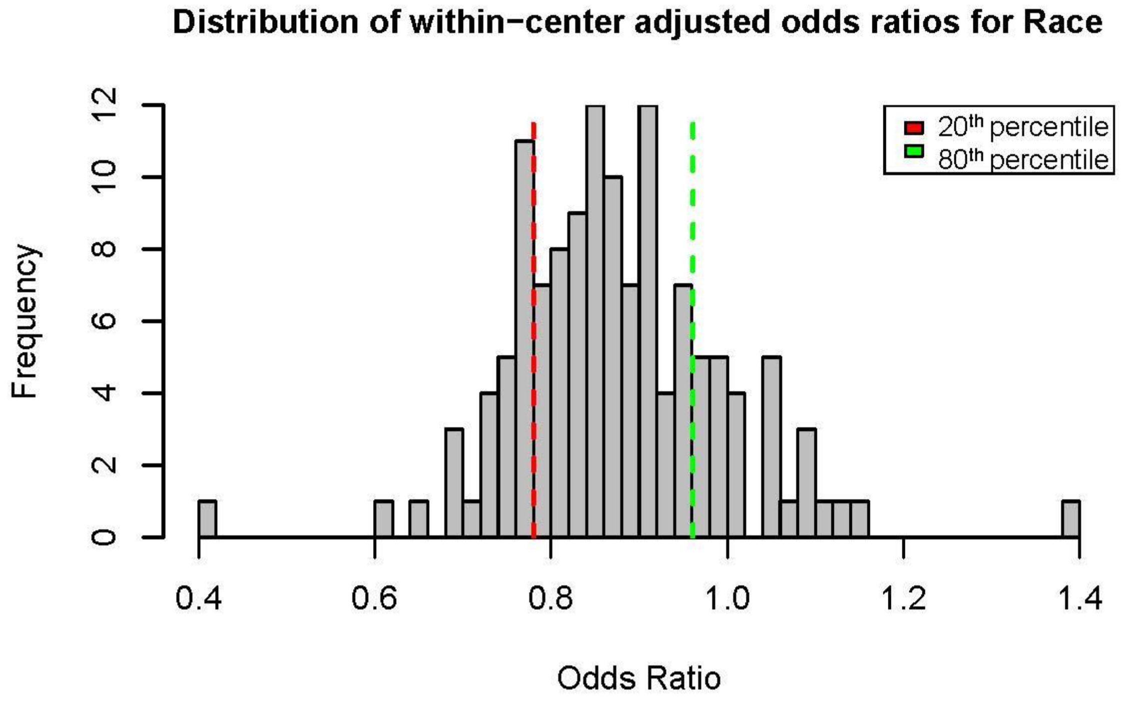

Hospitals in the lowest quintile (<20th percentile) are to the left of the red dashed line, and hospitals in the highest quintile (>80th percentile) are to the right of the green dashed line. The Odds Ratio reflects the odds of receiving Steroid treatment for COVID-19 for Black versus White patients. An odds ratio of less than 1 indicates a lower likelihood of Black patients receiving steroids than White patients.

© 2022 Castro AD et al. *JAMA Network Open.*

eFigure 4. Most Common Readmission Diagnoses Stratified by Race and Time Period

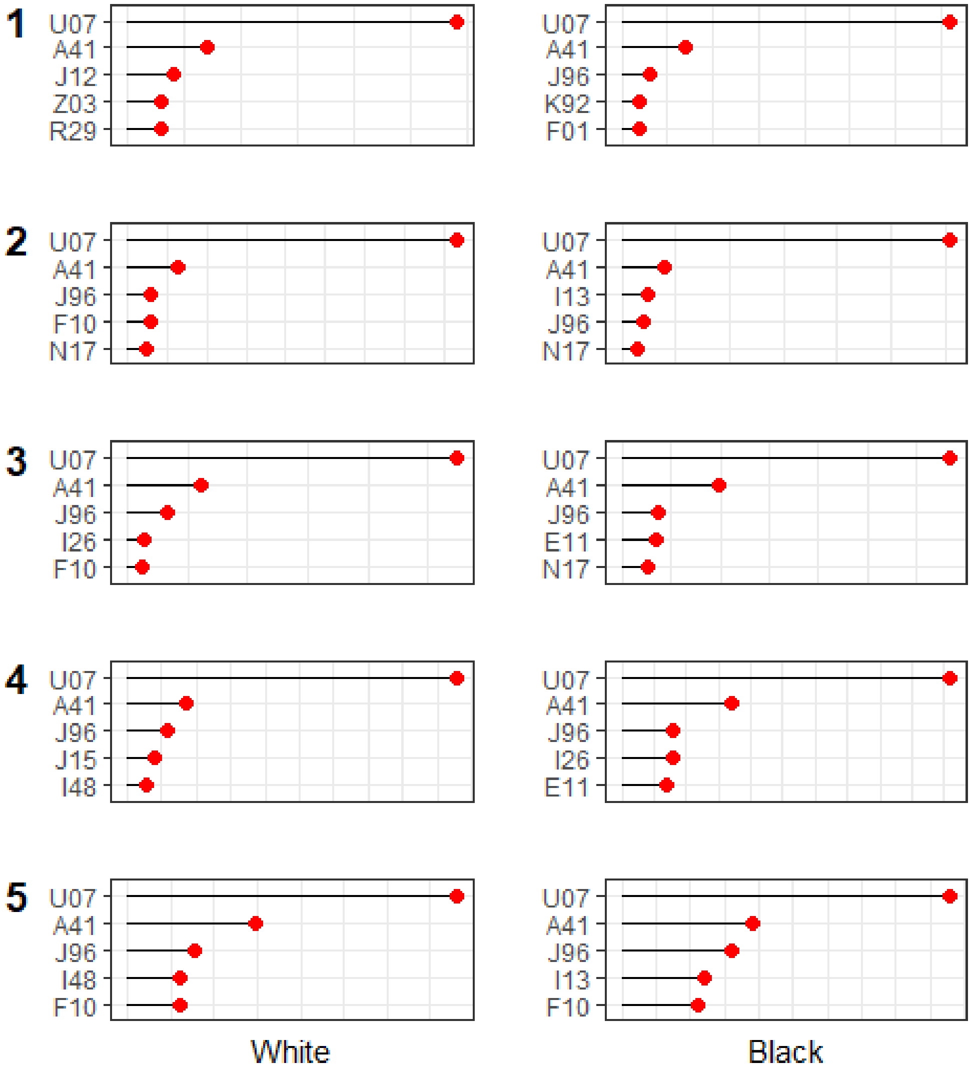

Period 1 included 170 readmissions between March 19 and August 6, 2020; Period 2 416 readmissions between May 23 and November 17, 2020; Period 3 2,297 readmissions between September 6, 2020, and December 2, 2021; Period 4 1,026 readmissions between June 14, 2021, and January 27, 2022; and Period 5 1,688 readmissions between November 4, 2021, and March 26, 2022.

[1]U07 Covid-19 disease
A41 Sepsis
J96 Respiratory failure
J15 Bacterial pneumonia
I26 Pulmonary embolism
I48 Atrial fibrillation and flutter
F10 Acute alcohol intoxication
I13 Hypertensive heart and renal disease
J12 Viral pneumonia
K92 Gastrointestinal hemorrhage
N17 Acute renal failure
E11 Diabetes mellitus

© 2022 Castro AD et al. *JAMA Network Open.*

eFigure 5. Balance Plot for Covariates Before and After Matching

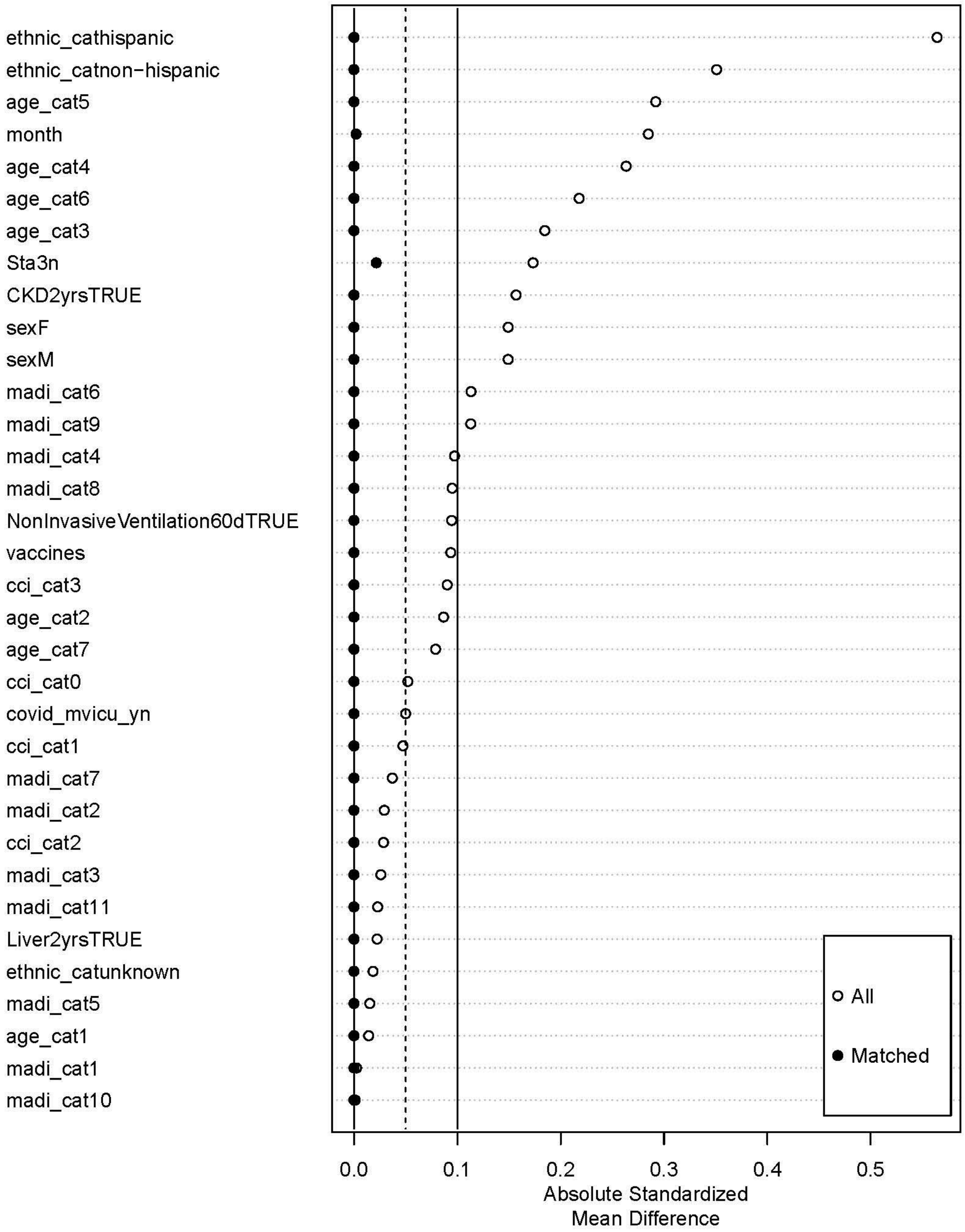

© 2022 Castro AD et al. *JAMA Network Open.*
